# An acetophenone carboxylase homolog from *Rhodococcus aetherivorans* initiates acetone rather than acetophenone catabolism

**DOI:** 10.3389/fmicb.2026.1888828

**Published:** 2026-10-05

**Authors:** Katharina Müller, Jörg Kahnt, Nicole Paczia, Johann Heider

**Affiliations:** 1 Microbial Biochemistry, Philipps-Universität Marburg, Marburg, Germany; 2 Proteomics Facility, Max-Planck-Institut für Terrestrische Mikrobiologie, Marburg, Germany; 3 Metabolomics Facility, Max-Planck-Institut für Terrestrische Mikrobiologie, Marburg, Germany

**Keywords:** acetone carboxylase, acetophenone carboxylase, acetophenone monooxygenase, ketone activation, *Rhodococcus*

## Abstract

Ketones such as acetone and acetophenone are chemically inert and must be activated prior to microbial degradation. In aerobic bacteria such as *Rhodococcus*, ketone activation is accomplished either by oxygenation or carboxylation reactions. Acetone and acetophenone carboxylases are homologous enzymes, but have different subunit compositions. While acetone carboxylases form heterohexameric complexes out of three distinct subunits, the acetophenone carboxylase core complex contains an additional duplicate of one of these subunits, resulting in a heterooctamer of four different subunits. Moreover, acetophenone carboxylase comprises a detachable fifth subunit ε, which is not present in acetone carboxylases. Here, we show that an apparent acetophenone carboxylase from *Rhodococcus aetherivorans* initiates acetone rather than acetophenone catabolism. The same strain of *R. aetherivorans* also degrades acetophenone, but as revealed by proteomics and enzyme activity measurements, this substrate is activated by an oxygenation reaction. In contrast to the canonical enzyme variant, which is restricted to acetone and butanone, the acetone carboxylase from *R. aetherivorans* apparently also accepts more aliphatic ketones with longer chain length. Furthermore, gene deletion studies confirmed the indispensability of the unique subunit ε for enzyme activity. In conclusion, we identified a novel type of acetone carboxylase with acetophenone carboxylase-like subunit composition and extended substrate spectrum. Phylogenetic analyses suggest that heterodecameric acetone carboxylases are particularly widespread across Actinomycetota and Pseudomonadota.

## 1 Introduction

Acetone and acetophenone are common intermediates of natural metabolism. For example, acetone is produced by Clostridia during solvent fermentation (Dürre et al., 1995), by methylobacteria as a product of polyhydroxybutyrate degradation (Thomson et al., 1976), or in leucine catabolism of *Vibrio* and *Pseudomonas* species (Massey et al., 1974; Nemecek-Marshall et al., 1999). Acetophenone is secreted by more than 20 plant families and some fungi to repel insects (Ahmadpourmir et al., 2024). In addition to their natural sources, acetone and acetophenone are released to the environment as industrial pollutants, e.g. as chemical solvents (Singh et al., 1994; Sanders et al., 1953).

As ketones such as acetone and acetophenone are chemically inert, they must be activated prior to microbial degradation. Under aerobic conditions, this is accomplished either by oxygenation or carboxylation (Ensign et al., 1998; Heider et al., 2016; Mus et al., 2020). Two different types of acetone oxygenations are known. First, acetone can be terminally hydroxylated to acetol. This reaction is catalyzed by a cytochrome P450 monooxygenase in mammals (Johansson et al., 1986; Koop and Casazza, 1985), by an analogous iron-dependent monooxygenase in mycobacteria (Furuya et al., 2011, 2015), and by a methane monooxygenase homolog in thermoacidophilic methanotrophs (Awala et al., 2021; Liu et al., 2025). Secondly, acetone may be activated to a methyl acetate ester. This reaction has been studied in the actinobacterium *Gordonia* sp. TY-5 and features a Baeyer-Villiger monooxygenase (Kotani et al., 2007). Members of the same enzyme class also initiate degradation of acetophenone and its derivatives in Actinomycetota such as *Arthrobacter* (Cripps, 1975) or *Nocardia* species (Cripps et al., 1978) and in Pseudomonadota, e.g., *Alcaligenes* (Higson and Focht, 1990) and *Pseudomonas* species (Tanner and Hopper, 2000). Here, acetophenone is oxygenated to a phenyl acetate ester, which is then hydrolyzed to acetate and phenol and further degraded via catechol (Havel and Reineke, 1993).

Acetone and acetophenone carboxylations are catalyzed by homologous enzymes from the hydantoinase/oxoprolinase family. Both proteins are highly conserved, but have different subunit compositions. While all known acetone carboxylases (Acx) were described as heterohexameric complexes out of three distinct subunits (Heider et al., 2016; Mus et al., 2020), the heterooctameric acetophenone carboxylase (Apc) core complex consists of four different subunits. Furthermore, Apc activity requires a fifth subunit (Apcε), which dissociates from the complex during purification and is not present in Acx (Jobst et al., 2010). Overall, the proposed reaction mechanisms of Acx and Apc are similar to each other. Both enzymes first activate both the ketone substrate and bicarbonate by phosphorylation. The phosphorylated intermediates are then transferred to a distinct catalytic metal-ion site on another subunit and react to the respective carboxylation products, acetoacetate or benzoylacetate. To catalyze the reaction, members of this carboxylase class do not require any organic cofactors, but different types of metal ions such as Zn^2+^, Fe^2+^, or Mn^2+^ (Boyd et al., 2004; Mus et al., 2017; Weidenweber et al., 2017; Jobst et al., 2010; Heider et al., 2016, Shisler et al., 2024; Mattice et al., 2025). Note that members of a biotin-dependent enzyme class carboxylate 4-hydroxyacetophenone in *Aromatoleum aromaticum* (Wöhlbrand et al., 2008), *Rhodococcus rhodochrous* GD02 (Dexter et al., 2022), *Sphingobium lignivorans* SYK-6 (Higuchi et al., 2022), and *Novosphingobium aromaticivorans* (Hall et al., 2025). However, it has been shown for both *A. aromaticum* and *R. rhodochrous* GD02 that these enzymes are not involved in acetophenone catabolism (Jobst et al., 2010; Dexter et al., 2022). Biotin-dependent acetone carboxylases are not known yet either.

While acetophenone carboxylase has only been studied in *A. aromaticum* so far (Jobst et al., 2010), several acetone carboxylases have been characterized in the literature. Most acetone carboxylases strictly depend on ATP hydrolysis and their substrate spectrum is limited to acetone and butanone (Sluis and Ensign, 1997; Schühle and Heider, 2012; Rosier et al., 2012). Intriguingly, a partially purified acetone carboxylase from *R. rhodochrous* B276 deviates from the other known enzymes. It hydrolyses inosine, guanosine, cytidine, uridine, and xanthosine triphosphates (ITP, GTP, CTP, UTP, and XTP) rather than ATP and its substrate spectrum extends to ketones with longer chain length (2-pentanone, 3-pentanone, 2-hexanone; Clark and Ensign, 1999). As *R. rhodochrous* B276 has not been sequenced and the strain is not available, we chose the phylogenetically related actinobacterium *Rhodococcus aetherivorans* for further investigations on this enzyme.

## 2 Results

### 2.1 *Rhodococcus aetherivorans* encodes an apparent acetophenone carboxylase homolog and grows both on acetone and acetophenone

We screened the genome of *R. aetherivorans* for the presence of putative *acx* or *apc* operons. Thereby, we identified only one homologous gene cluster, which included five genes and thus resembled the *A. aromaticum* acetophenone carboxylase operon (five genes; Jobst et al., 2010) rather than the canonical acetone carboxylase operons (three genes; Heider et al., 2016). In agreement with this, four of the encoded proteins shared higher sequence similarity with *A. aromaticum* Apc than with Acx subunits. The fifth encoded subunit did not share significant sequence similarity with *A. aromaticum* Apcε, but had a comparable size of approximately 30 kDa (Figure 1A). We hypothesized that the operon encodes an unusual enzyme variant in *Rhodococcus* species, which may carboxylate not only acetophenone, but also acetone, accounting for the unique biochemical properties of the *R. rhodochrous* enzyme reported in the literature (Clark and Ensign, 1999). To verify our hypothesis, we first tested growth of *R. aetherivorans* on acetone and acetophenone. Indeed, the bacterium was capable of utilizing both of these substrates (Figure 1B), exhibiting growth rates of 0.24 ± 0.03 h^–1^ on acetone and 0.11 ± 0.01 h^–1^ on acetophenone.

**FIGURE 1:**
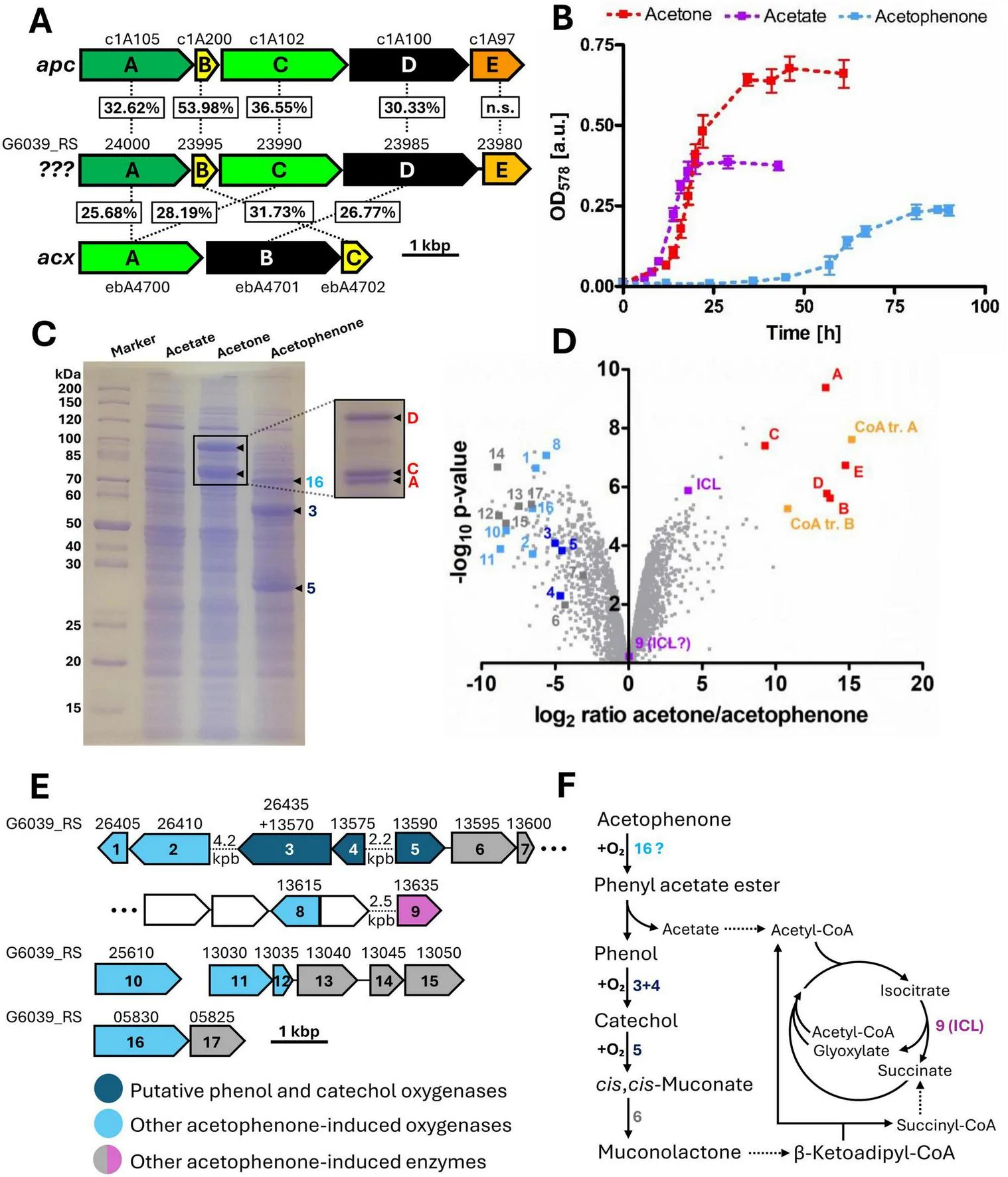
Different mechanisms for acetone and acetophenone activation in *Rhodococcus aetherivorans*. **(A)** Putative *apc*/*acx* gene cluster from *R. aetherivorans* (middle row) in comparison to *Aromatoleum aromaticum apc* (upper row) and *acx* homologs (bottom row). NCBI locus tags are provided. The percentages indicate protein sequence identities. The subunits A and C encoded in the five-gene operons are homologous to each other and are therefore shown in similar green tones. n.s., non-significant. **(B)** Growth of *R. aetherivorans* on acetone, acetate, and acetophenone. **(C)** SDS-PAGE (10%) of extracts from cells grown on acetate, acetone, and acetophenone (20 μg protein loaded per lane). Bands marked with an arrow were analyzed by mass spectrometry (Supplementary Table 1). Small window: Excerpt from a 7% SDS polyacrylamide gel in which the same extract from acetone-grown cells has been separated (5 μg protein loaded). **(D)** Whole cell proteomics results of *R. aetherivorans* during growth on acetone vs. acetophenone. For raw data, see Supplementary Table 2. The *p*-values were calculated using Welch’s *t*-tests. **(E)** Acetophenone-induced gene clusters, which are potentially involved in acetophenone catabolism. Gene 3 from the upper operon is split over two contigs and incomplete; gene length was inferred from OCS65_RS03735 (WP_263508839.1, *R. aetherivorans* N1). **(F)** Apparent acetophenone catabolic pathway. Numbers correspond to the labels in panels **(C–E)**. UniProt Annotations of enzyme functions: 1 and 2, Benzoate 1,2-dioxygenase small and large subunit; 3, Pyoverdin chromophore biosynthetic protein pvcC; 4, Flavin reductase family protein; 5, Catechol 1,2-dioxygenase; 6, Muconolactone Delta-isomerase; 7, Muconate/chloromuconate family cycloisomerase; 8, 4,5-DOPA dioxygenase extradiol; 9, Isocitrate lyase/phosphoenolpyruvate mutase family protein; 10 and 11, Propane 2-monooxygenase; 12, MmoB/DmpM family protein; 13, Amidohydrolase; 14, Iron-sulfur cluster assembly protein; 15, NAD(P)-dependent alcohol dehydrogenase; 16, NAD(P)/FAD-dependent oxidoreductase; 17, Alpa/beta hydrolase.

### 2.2 The Apc homolog only initiates acetone catabolism, while acetophenone is activated by an oxygenase

As expected, the five subunits of the Apc homolog were strongly induced by acetone. The three large subunits (71, 77, and 85 kDa) were clearly visible in cell extracts separated by SDS-PAGE. Notably, two of the subunits (71 and 77 kDa) migrated close to each other and could only be separated in gels with very low (7%) acrylamide concentrations (Figure 1C and Supplementary Table 1). The three large subunits together accounted for about 11% ± 1.5% of the total soluble protein in acetone-grown cells. Moreover, whole cell proteomics revealed strong induction of CoA transferases and isocitrate lyase in acetone-grown cells (Figure 1D and Supplementary Table 2). This is in line with the canonical pathway for acetone degradation: Acetone carboxylation yields acetoacetate, which is activated to its CoA ester, potentially by a CoA transferase. Acetoacetyl-CoA is then thiolytically cleaved into two molecules of acetyl-CoA and shuttled into the glyoxylate cycle (Schühle and Heider, 2012). Notably, acetone-induced expression of the apparent *apc* operon was observed not only in minimal medium, but also during growth in LB medium supplemented with acetone (Supplementary Table 3), indicating that the operon is not regulated by catabolite repression.

To our surprise, the Apc homolog was not present in acetophenone-grown cells. Instead, two operons harboring multiple annotated oxygenase genes were induced by acetophenone, suggesting that acetophenone is activated via oxygenation. The acetophenone-induced operons included genes for annotated phenol and catechol oxygenases as well as a putative isocitrate lyase (Figures 1C–E and Supplementary Tables 1, 2). This is in accordance with the expected downstream catabolic pathway (Figure 1F; Havel and Reineke, 1993).

Next, we wanted to confirm the proposed acetone carboxylase and acetophenone oxygenase activities in the respective cell extracts. To explain the observed growth rates, we expected specific substrate assimilation rates of ≥160 mU mg protein^–1^ for acetone carboxylase and ≥40 mU mg protein^–1^ for acetophenone monooxygenase (see section “4 Materials and methods” for calculations). Acetone carboxylase was measured using a coupled spectrophotometric assay, in which the carboxylation product acetoacetate was reduced to 3-hydroxybutyrate by an NADH-dependent auxiliary enzyme, 3-hydroxybutyrate dehydrogenase. The measured activities of 3.7 ± 1.9 mU mg^–1^ protein were lower than expected and showed strong batch-dependent variation. To our surprise, we also observed acetone-dependent NADH oxidation in acetophenone-grown cells (Table 1), although none of the acetone carboxylase subunits had been detected in previous proteomics experiments (Figure 1D). Therefore, we performed additional LC-MS assays with acetone as a substrate and showed that acetoacetate was formed only in extracts from acetone-grown cells, but not in those of cells grown on acetophenone (Supplementary Figure 1).

**TABLE 1:** Enzymes of acetone and acetophenone catabolism in *R. aetherivorans*.

Enzyme	Cofactors	Specific activity [nmol min^–1^ mg^–1^ protein]		Candidate gene(s)	
		Acetone	Acetophenone	NCBI accession(s) G6039_RS	Label(s) in Figures 1C–F
Acetone carboxylase	ITP	3.7 ± 1.9 (*n* = 5)	5.7 ± 3.2 (*n* = 3)	24000 23995 23990 23985 23980	A B C D E
Isocitrate lyase		140 ± 16 (*n* = 3)	21.7 ± 8.9* (*n* = 2) [10.9 ± 4.4]	16640 13635	ICL 9
Acetophenone monooxygenase	NADPH	<0.1 (*n* = 3)	8.3 ± 4.3 (*n* = 5)	05830	16
	NADH	ND	<1 (*n* = 2)		
Phenol hydroxylase	NADPH	ND	<0.4* (*n* = 2)	26435 + 13570/13575	3/4
	NADH	ND	1.0 ± 0.6* (*n* = 2) [0.5 ± 0.3]		

Most activities were measured at 28 °C. Where indicated with an asterisk (*), assays were performed at 38 °C; activities extrapolated to 28 °C (i.e., measured activities divided by two) are shown in brackets. The numbers of biological replicates *n* are indicated in the table. For each biological replicate, at least two technical replicates were carried out. Errors refer to the standard deviations of the biological replicates. ND, not determined.

Acetophenone monooxygenase activity was detected in extracts from acetophenone-grown cells, but was absent in cells grown on acetone. The reaction was NADPH-dependent (Table 1). Furthermore, we observed substrate-independent, uncoupled NADPH oxidation at 2.6-fold lower rates (Supplementary Table 4). Hence, to calculate activities, we subtracted background activity values obtained without substrate from the activities measured with acetophenone. Finally, we measured two of the downstream catabolic enzymes. We confirmed isocitrate lyase activities in extracts from both acetone- and acetophenone-grown cells. Also, we observed very low, NADH-dependent phenol hydroxylase activity in extracts from acetophenone-grown cells (Table 1).

### 2.3 The Apc homolog accepts ketones with longer chain length, but not acetophenone

To analyze the substrate spectrum of the enzyme, we performed enzyme assays with extracts from acetone-grown cells and different ketones as organic substrates. Thereby, we employed inosine triphosphate (ITP) as nucleotide cofactor because the previously studied acetone carboxylase from *R. rhodochrous* exhibits a preference for ITP and does not accept ATP (Clark and Ensign, 1999). Next, we conducted untargeted high resolution mass spectrometry measurements to test for the formation of the respective carboxylation products, probably catalyzed by the Apc homolog studied here. Intriguingly, we detected the carboxylation products of not only acetone and butanone, but also of 2-pentanone, 3-pentanone, and 2-hexanone (Figure 2). This was in accordance with our expectations as the acetone carboxylase from *R. rhodochrous* shows the same substrate specificity (Clark and Ensign, 1999). In contrast, acetophenone was not carboxylated in the extract. As the novel acetone carboxylase from *R. aetherivorans* seems to differ substantially from conventional acetone carboxylases and is unable to convert acetophenone, we will refer to it as Acr henceforth (“acetone carboxylase from *Rhodococcus*”).

**FIGURE 2:**
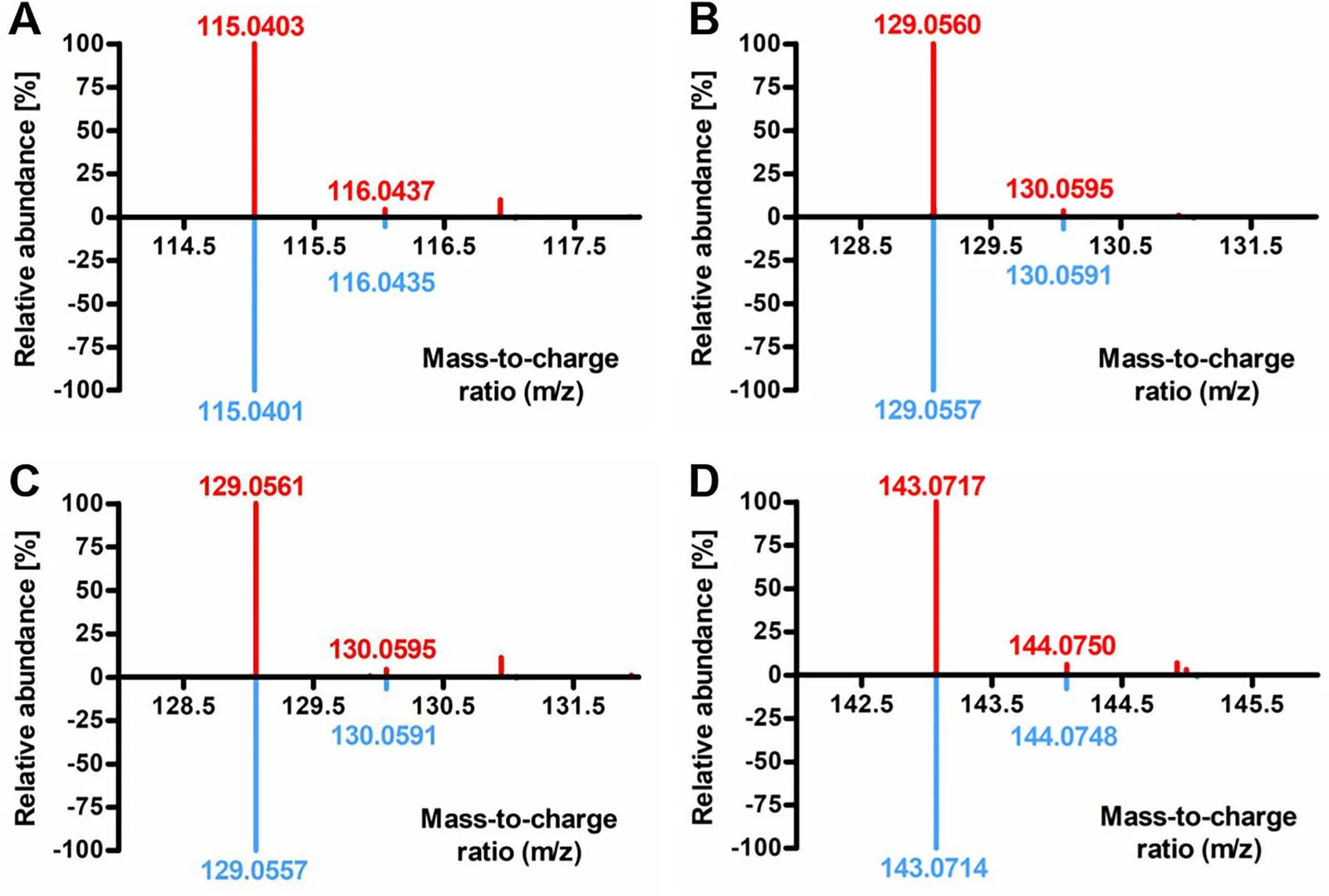
Acr-catalyzed carboxylation of panel **(A)** 2-butanone, **(B)** 2-pentanone, **(C)** 3-pentanone, and **(D)** 2-hexanone. LC-MS data are displayed as relative ion counts against mass-to-charge ratio (m/z). Spectra were extracted at the apex of the respective extracted ion chromatogram (EIC) peaks generated for the theoretical m/z values of each carboxylation product, corresponding to retention times of panel **(A)** 5.721–7.132 min (3-oxopentanoate), **(B)** 7.396–7.563 min (3-oxohexanoate), **(C)** 7.364–7.519 min (4-oxohexanoate), and **(D)** 7.638–8.016 min (3-oxoheptanoate). Each experimental spectrum (red) is shown as a mirror plot against the corresponding theoretical isotope pattern (blue) calculated based on natural isotope abundance. All assays were performed in duplicates and the duplicate spectra were comparable to each other. No carboxylation products of the relevant masses were detected with acetophenone or with the water control as substrates.

### 2.4 The unique subunit ε is indispensable for acetone carboxylase activity

Next, we wanted to gain additional evidence for the involvement of the unique subunit ε in enzyme activity. Therefore, we generated an *acrE* deletion mutant of *R. aetherivorans* and tested its growth on acetone versus acetate or acetophenone. As expected, the deletion mutant showed an acetone-specific growth defect (Figure 3), indicating that the unique subunit ε is indeed indispensable for acetone carboxylation. Note that complementation of the deletion mutant with a cloned *acrE* gene did not succeed. To rule out potential effects of *acrE* deletion on expression of the genes for the other four subunits or the transcriptional regulator within the same operon, we cultivated wildtype and *acrE* deletion mutant in LB medium containing 10 mM acetone and then performed whole-cell proteomics. All of the Acr subunits were expressed at comparable levels in wildtype and deletion mutant, except for Acrε, which was detected only in the wildtype (Supplementary Table 3). Intriguingly, when we analyzed the proteomics data in more detail, we identified a peptide corresponding to an N-terminal extension of the annotated open reading frame of Acrε. Indeed, we observed in-frame CTG and GTG codons upstream of the annotated *acrE* gene (Supplementary Figure 2), which may serve as alternative start codons, providing a possible explanation for the failed complementation experiment.

**FIGURE 3:**
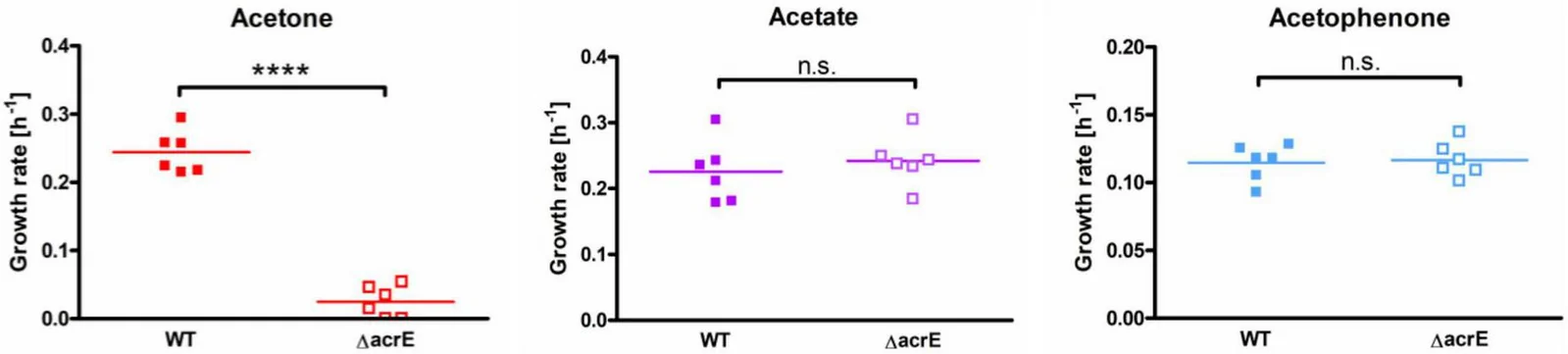
Indispensability of the unique subunit ε for acetone carboxylation. Growth rates of *R. aetherivorans* WT and Δ*acrE* on acetone, acetate, or acetophenone. The following *p*-values were calculated using Student’s *t*-tests in Microsoft Excel (two-tailed distribution, two-sample equal variance): acetone, 7.58e-08; acetate, 0.80; acetophenone, 0.53. n.s, non-significant (*p* > 0.05). ****p < 0.0001.

### 2.5 Heterodecameric acetone carboxylases mainly occur in Actinomycetota and Pseudomonadota

Finally, we studied the distribution of heterodecameric ketone carboxylases across microbes. In our phylogenetic analysis, we first searched for the small Apcβ/Acxγ homologs because these subunits are specific to Apc and Acx. In contrast, homologs of the large subunits are additionally found in tetrameric proteins from the hydantoinase/oxoprolinase superfamily (Ogawa et al., 1995; Kumar and Bachhawat, 2010; Marjanovic et al., 2021). Then, we screened for the presence of the corresponding genes within *acr/apc*-like operons and constructed a phylogenetic tree based on an alignment of putative subunits ε (Figure 4). Intriguingly, these subunits clustered in different groups, which were highly divergent from each other and showed only minor or even no detectable pairwise sequence identity. Close homologs of the subunit Acrε from *R. aetherivorans* occur in only a few different (phylogenetically closely related) species and in several non-classified strains of *Rhodococcus* (in total, encoded in >90 genomes; NCBI-refseq database). Specifically, these proteins are present in all sequenced *R. aetherivorans* strains (in total, 26), in 90% of sequenced *R. ruber* strains (38 out of 42), as well as in *R. zopfii* (5 out of 6 genomes) and *R. indonesiensis* (2 out of 3 genomes), and thus seem to be characteristic for these species. For *R. rhodochrous*, however, Acr is not a typical feature since it is encoded in only 20% of the sequenced strains (5 out of 26). Importantly, in our analysis we did not find canonical *acx* operons in any sequenced *Rhodococcus* strain. Apart from *Rhodococcus* strains, *acr* gene clusters encoding for close homologs of Acrε are present in about 150 other actinobacterial genomes and in several metagenome assemblies (NCBI-refseq database). Hence, Acr is not particularly widespread, but it occurs in several different actinobacterial genera, including *Arthrobacter* (5 species), *Mycobacterium* and *Mycolicibacterium* (14 species), *Cryobacterium* (11 species), and further, minor-represented genera. In addition, it seems that the *acr* gene cluster has occasionally been transferred horizontally from Actinomycetota to Pseudomonadota, e.g. *Marinobacter gelidimuriae* (Figure 4).

**FIGURE 4:**
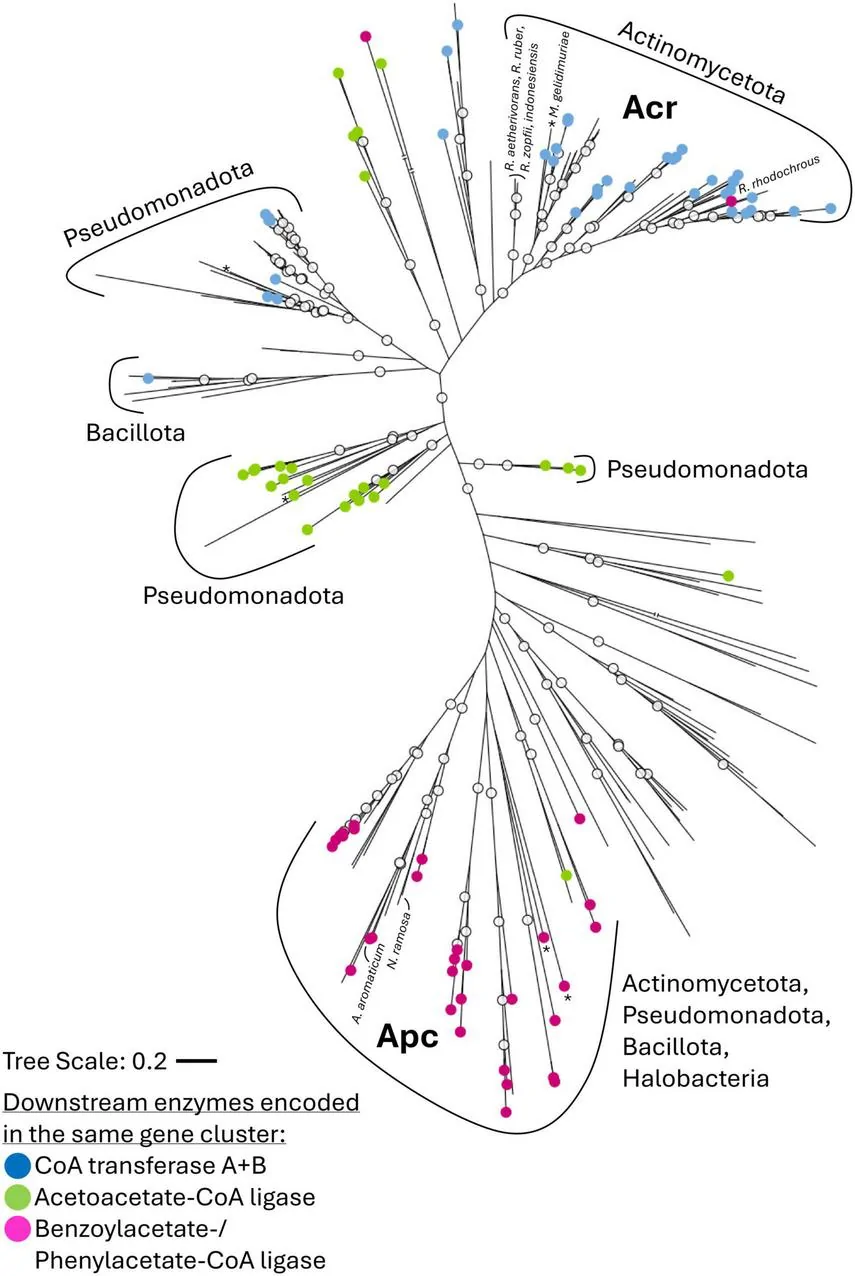
Phylogenetic tree of subunits ε from heterodecameric ketone carboxylases. Apcβ/Acxγ homologs were identified (Supplementary Table 5), the corresponding genes were screened for their presence within an *apc*-like operon (i.e., encoding three hydantoinase/ oxoprolinase homologs, one Apcβ/Acxγ homolog, and one additional hypothetical protein of ca. 30 kDa), and the sequences of the hypothetical proteins (putative Apcε homologs) were aligned via MUSCLE (Edgar, 2004). The phylogenetic tree was constructed using the Maximum Likelihood algorithm with the Jones-Taylor- Thornton model (Jones et al., 1992) implemented in MEGA12 (Kumar et al., 2024). Bootstrapping was performed in MEGA12 with 500 bootstrap replicates. Bootstrap values ≥ 0.7 are displayed in the figure. iTOL (Letunic and Bork, 2007) was used for visualization. The scale bar represents 0.2 amino acid substitutions per site. Marked with an asterisk (*): other phylogenetic classification than indicated.

Branches of heterodecameric carboxylases with more distantly related subunits ε mainly occur in Alpha-, Beta-, and Gammaproteobacteria (encoded in 30–45 genomes each), with *Xanthobacter* sp., *Burkholderia* sp., and *Azotobacter* sp. being by far the most prominent genera (≥12 genomes each), and in a few Bacillota (8 genomes). The presence of downstream genes for acetoacetate-converting enzymes in both the actinobacterial and the proteobacterial operons suggests that both operons encode acetone rather than acetophenone carboxylases. In contrast, a branch of five-gene operons including the gene for *A. aromaticum* Apcε probably encodes acetophenone carboxylases. Indeed, several of these operons cluster with genes coding for homologs of a putative benzoylacetate-CoA ligase from *A. aromaticum* (Figure 4), which has been proposed to activate the acetophenone carboxylation product (benzoylacetate) to its CoA ester for further assimilation (Rabus et al., 2002).

### 2.6 The subunits ε from heterodecameric acetone and acetophenone carboxylases are highly divergent, but evolved from a common ancestor protein

Intriguingly, Acrε from *R. aetherivorans* and Apcε from *A. aromaticum* did not share any detectable sequence identity, while the other four subunits encoded by the respective gene clusters were clearly homologous (Figure 1A). We therefore asked whether the subunits ε from heterodecameric acetone and acetophenone carboxylases evolved from a common ancestor protein and created a Hidden Markov model with all *R. aetherivorans* Acrε homologs detectable in the NCBI non-redundant protein database. While the model did not identify Apcε from *A. aromaticum* as a putative homolog, the sequences of other apparent Apcε proteins from the same phylogenetic branch were recovered (e.g., Apcε from *Nevskia ramosa*, *E*-value 7.6e-06; Supplementary Table 6). In addition, a Hidden Markov model trained with all detectable homologs of Apcε from *A. aromaticum* recovered the sequence of Acrε from *R. aetherivorans* (*E*-value 4e-06; Supplementary Table 7). We conclude that the subunits ε from heterodecameric acetone and acetophenone carboxylases are highly divergent, but evolutionary related proteins. Moreover, as predicted by AlphaFold, they still show structural similarity (Supplementary Figure 3) and according to our Foldseek analysis, the structural alignment of the two proteins is statistically significant (*E*-value 5e-07). In accordance with these results, a phylogenetic tree of Apcβ/Acxγ homologs (Supplementary Figure 4) looked similar to the phylogenetic tree of subunit ε (Figure 4), indicating a shared evolutionary origin of these subunits within their respective operons.

## 3 Discussion

### 3.1 Acetone and acetophenone catabolism in *R. aetherivorans*

In this study, we show that the acetophenone carboxylase homolog from *R. aetherivorans* (Acr) initiates acetone rather than acetophenone catabolism. In contrast, acetophenone is activated by an oxygenation reaction. The respective (candidate) enzymes are strongly induced in acetone- and acetophenone-grown cells (Figure 1), which may be regarded as good evidence that they actually catalyze the first reactions of the respective pathways: They probably have to be highly abundant in the living cells even under physiological conditions to enable the required metabolic fluxes. Despite their high abundance, the observed acetophenone monooxygenase and acetone carboxylase activities (Table 1) were roughly 7- and 43-fold lower than the respective expected values. Moreover, acetone carboxylase activity was 10-fold lower than the specific activity values reported for *Aromatoleum aromaticum* Acx, although *A. aromaticum* exhibits lower growth rates on acetone than *R. aetherivorans* (Schühle and Heider, 2012). This discrepancy may be explained by technical limitations. Cell disruption of gram-positives such as *R. aetherivorans* is challenging: when conditions are too mild, cells are not disrupted properly and protein concentrations are too low to measure activities. On the other hand, when conditions are too harsh, enzymes may get inactivated or large complexes disrupted. These technical issues are particularly relevant for the five-subunit complexes of Acr and Apc, compared to the more compact three-subunit variants of Acx. Indeed, Apc has been described previously to exhibit notorious instability (Heider et al., 2016; Weidenweber et al., 2017) and intrinsically low specific activity (Jobst et al., 2010; Schühle and Heider, 2012). Standard deviations of activity measurements were high (Table 1), again probably due to varying degrees of enzyme inactivation during cell disruption (e.g., dissociation of subunit ε from the Acr complex or loss of FAD from oxygenases). In addition, there may be real differences in enzyme abundance or integrity. Indeed, Acr expression levels clearly varied across different cell batches (Supplementary Figure 5).

As Acr is not present during growth on acetophenone (Figures 1C,D and Supplementary Tables 1, 2), the observed acetone-dependent NADH oxidation in acetophenone-grown cells remains elusive (Table 1). We hypothesize that some of the acetophenone-induced oxygenases may exhibit side activity toward acetone. Further work is required to characterize the substrate range and other biochemical properties of the acetophenone-induced oxygenases. While acetophenone oxygenation was unambiguously detected in extracts of acetophenone-grown cells, phenol hydroxylase activity was observed only at low levels barely above the detection limit (Table 1). However, proteomic analysis provided convincing evidence for the presence of a phenol hydroxylase in acetophenone-grown cells because the acetophenone-induced candidate enzyme in *R. aetherivorans* (Figures 1C–E) shares high sequence identity with an experimentally confirmed phenol hydroxylase from *Rhodococcus erythropolis* (NCBI protein accessions ABS30825.1 and ABS30826.1, Saa et al., 2010, Supplementary Figure 6). Finally, the low specific activities (Table 1) are consistent with the strong induction of the putative phenol hydroxylase gene in acetophenone-grown cells (Figures 1C,D and Supplementary Tables 1, 2).

### 3.2 Acetophenone carboxylase-like subunit composition of *Rhodococcus* acetone carboxylase

We propose that *R. aetherivorans* Acr represents a novel type of acetone carboxylase. As opposed to the canonical heterohexameric acetone carboxylases (Sluis and Ensign, 1997; Sluis et al., 2002; Schühle and Heider, 2012; Rosier et al., 2012), Acr has an acetophenone carboxylase-like subunit composition and consists of five rather than three distinct subunits (Figures 1, 3). The structural difference appears to be accompanied by an extended substrate spectrum (Figure 2). While canonical acetone carboxylases form an intramolecular channel to transport the phosphorylated substrates from the activation site to a distinct catalytic center (Mus et al., 2017), such a channel is not present in the octameric Apc core complex. Instead, it has been proposed that the detachable subunit Apcε protects the activated intermediates from hydrolysis during the transport from the activation to the catalysis site (Weidenweber et al., 2017). The high structural similarity with Apc suggests that a similar mechanism operates in Acr. In this scenario, substrate size of Acr would not be limited by the dimensions of a putative intramolecular channel, which might explain the extended substrate range compared to canonical acetone carboxylases (i.e., carboxylation of ketones with longer chain length). To gain further insights into substrate transport and the role of subunit ε therein, the structure of the Acr complex should be elucidated. Determination of the protein structure would also help to better understand the functional differences between Acr and Apc.

Remarkably, a previous study on the acetone carboxylase from the closely related strain *R. rhodochrous* B276 claimed that the partially purified enzyme consisted of only three subunits (85, 74, and 16 kDa). However, we demonstrate here that two of the subunits of *R. aetherivorans* Acr tend to comigrate in SDS-PAGE analysis, especially in gels with high acrylamide concentrations (Figure 1C). This probably resulted in a misinterpretation of the subunit pattern in the former study. Furthermore, the reported size of the small subunit of the *R. rhodochrous* enzyme (16 kDa) is rather small compared to the size of canonical Acxγ subunits (19–20 kDa; Sluis and Ensign, 1997; Sluis et al., 2002; Schühle and Heider, 2012; Rosier et al., 2012), but matches the sizes of *A. aromaticum* Apcβ (15 kDa; Jobst et al., 2010) and Acrβ from the enzyme studied here (16.6 kDa). Therefore, the acetone carboxylase from *R. rhodochrous* B276 most probably belongs to the novel type of acetone carboxylase, too. Although *R. rhodochrous* B276 has unfortunately not been sequenced, a highly conserved *acr* operon is present in several other *R. rhodochrous* strains. In fact, the protein sequences of *R. aetherivorans* and *R. rhodochrous* Acr subunits share sequence identities of ≥41%. In conclusion, our study provides an explanation for the biochemical differences between the acetone carboxylases from *Rhodococcus* (Clark and Ensign, 1999; this study) and Pseudomonadota (Sluis and Ensign, 1997; Schühle and Heider, 2012; Rosier et al., 2012).

### 3.3 Phylogenetic distribution of the novel type of acetone carboxylase and alternative oxygenases for acetone activation

The novel type of acetone carboxylase studied here mostly occurs in Actinomycetota and is characteristic for (but not limited to) certain *Rhodococcus* species. Furthermore, our phylogenetic analysis suggests that other (phylogenetically more distant) branches of heterodecameric acetone carboxylases are present in Pseudomonadota (Figure 4). Future studies are required to test the actual acetone carboxylation activity of the proteobacterial enzyme variants and to compare the different enzymes in terms of biochemistry.

Most Actinomycetota are strictly aerobic and would in principle be able to employ oxygenation as alternative strategy for acetone activation. Although we cannot predict the distribution of acetone oxygenases based on sequence identity, several actinobacterial strains have been reported to possess such an enzyme (Kotani et al., 2007; Cripps, 1975; Cripps et al., 1978). As indicated by the acetone oxygenase side reactivities observed in acetophenone-grown cells (Table 1), the strain studied here may have the genetic potential for acetone oxygenation, too. However, this putative acetone oxygenase is obviously not involved in acetone catabolism (Figure 3), either because it is not induced by acetone or due to a lack of suitable downstream catabolic enzymes. Which ketone activation strategy is more favorable? On the one hand, acetone carboxylation is very energy-intensive because it requires high production levels of the respective proteins (Figures 1C,D and Supplementary Tables 1, 2) and two nucleotide triphosphates are hydrolyzed per reaction (Heider et al., 2016). However, energy is usually not the growth-limiting factor for aerobes. Oxygenation reactions, on the other hand, may generate reactive oxygen species, which damage the cells. This requires strong expression of detoxifying enzymes such as catalase, as observed in acetophenone-grown cells in this study (Supplementary Tables 1, 2). Moreover, oxygenases are energy-demanding, too, not only due to their low activities (Table 1) and strong induction (Figures 1C,D and Supplementary Tables 1, 2), but also since they consume reducing equivalents independently of substrate activation (Supplementary Table 4).

### 3.4 Limitations of cell extract studies

Carboxylation of acetone, butanone, 2-pentanone, 3-pentanone, and 2-hexanone has been unambiguously detected in extracts from acetone-grown *R. aetherivorans* by LC-MS (Figure 2, Supplementary Figure 1). We attribute these activities to the novel acetone carboxylase, but measurements with purified enzyme are required to confirm this. Assays were conducted with ITP as nucleotide cofactors and cell extracts were depleted of endogenous nucleotides by ultrafiltration prior to the measurements. As expected, we did not observe acetone carboxylation in ITP-depleted control measurements (Supplementary Figure 7). However, enzymes in the cell extract may still catalyze the conversion of ITP into triphosphates of any other residual nucleotides, including ATP. Hence, confirmation (or disproof) of the Acr dependency on ITP or other nucleotide triphosphates except ATP needs to be performed with purified enzyme. Purification of the enzyme is also necessary for further characterization, e.g., metal analysis and determination of enzyme kinetics with different substrates.

### 3.5 Biotechnological potential of *R. aetherivorans* acetone carboxylase and its promoter

*Rhodococcus aetherivorans* Acr is strongly induced by acetone even during growth in LB medium (Supplementary Table 3). This has interesting biotechnological implications. For instance, the promoter of *R. aetherivorans* Acr may be used for inducible heterologous protein production. Indeed, *Rhodococcus* species currently emerge as expression platform for actinobacterial proteins. These (sometimes toxic) proteins are encoded by genes with high GC contents, which usually cannot be expressed in *E. coli* without codon optimization (Ngo et al., 2025). Finally, putative biotechnological applications of *R. aetherivorans* acetone carboxylase in e.g. synthetic pathways need to be evaluated after purification and further characterization of the novel enzyme.

## 4 Materials and methods

### 4.1 Materials

Chemicals were purchased from Alfa-Aesar, Applichem, Cayman, Fluka, Fluorochem, Merck, Roth, TCI, Thermo Fisher Scientific, or VWR Life Sciences. Materials for molecular biology were obtained from Thermo Fisher Scientific. Primers were synthesized by Microsynth. pDD57 plasmids for complementation experiments were initially provided by Anna C. Ngo (AG Tischler, Bochum) and later re-ordered from AddGene (#119882, DeLorenzo et al., 2018).

### 4.2 Strains and growth conditions

*Rhodococcus aetherivorans* 10BC-312 (DSM 44752) was obtained from the Deutsche Sammlung von Mikroorganismen und Zellkulturen (DSMZ). Growth of bacteria was monitored by optical density measurements at 578 nm. *R. aetherivorans* was cultivated aerobically at 28 °C either on agar plates (15 g l^–1^ agar) or on a rotary shaker (130 rpm) in LB medium or minimal medium. Where indicated, the LB medium was supplemented with 10 mM acetone. The minimal medium (pH 8.0) contained 3.67 mM KH_2_PO_4_, 5.61 mM NH_4_Cl, 2.03 mM MgSO_4_⋅7H_2_O, 0.68 mM CaCl_2_⋅2H_2_O, 3.53 mM NaNO_3_, 40 mM NaHCO_3_, 1 ml l^–1^ trace element solution SL-10 (10 ml l^–1^ 25% HCl, 1.5 g l^–1^ FeCl_2_⋅4H_2_O, 70 mg l^–1^ ZnCl_2_, 100 mg l^–1^ MnCl_2_⋅4H_2_O, 6 mg l^–1^ H_3_BO_3_, 190 mg l^–1^ CoCl_2_⋅6 H_2_O, 2 mg l^–1^ CuCl_2_⋅2H_2_O, 24 mg l^–1^ NiCl_2_⋅6H_2_O, and 36 mg l^–1^ Na_2_MoO_4_⋅2H_2_O), and 10 mM of an organic carbon source (potassium acetate, acetophenone, or acetone). Trace element solution, potassium acetate, and NaHCO_3_ were prepared in separate solutions and added after autoclaving. Cultivation was performed in butyl rubber-sealed flasks to prevent escape of CO_2_ and evaporation of solvents. The flasks were filled to only 30% of their volume to ensure sufficient oxygen supply for aerobic growth.

*Escherichia coli* DH5α and WM 3064 were grown at 37 °C in lysogeny broth medium. For diaminopimelate (DAP)-auxotrophic *E. coli* strain WM 3064, the medium was supplemented with 0.1 mM or 0.25 mM DAP (solid or liquid media, respectively). Antibiotics were added to the cultures to final concentrations of 100 μg ml^–1^ (ampicillin), 50 μg ml^–1^ (kanamycin), or 20 μg ml^–1^ (gentamicin).

### 4.3 Harvesting of cells

*Rhodococcus aetherivorans* cultures were harvested in the exponential growth phase (OD 0.2–0.4 when inoculating to a starting OD of 0.01). Cells were pelleted by centrifugation (2,370 × *g*, 4 °C, 15 min). Then, cells were washed with ice-cold 0.9% saline twice, followed by centrifugations (17,000 × *g*, 4 °C, 15 min). Cell pellets were stored at −80 °C until further use.

### 4.4 Preparation of cell extracts

Frozen cells were washed with ice-cold 0.9% saline three times, followed by centrifugations (17,000 × *g*, 4 °C, 2 min). Cell pellets were resuspended in a 1:2 (w/v) volume of resuspension buffer (20 mM Tris-HCl, pH 8.0, 5 mM DTE, 0.4 mg ml^–1^ lysozyme, 1% Triton X-100) and incubated at room temperature for 10–15 min. During the incubation period, the mixture was vortexed several times. To promote physical cell disruption, 3–8 glass beads (1 mm diameter) per ml resuspended cells were added to the suspension. Then, cells were lysed by sonication using a Bandelin SONOPULS device (30% amplitude, 1 s pulse, 2 s break, 3–6 min). During sonication, cells were kept on ice. Cell debris was removed by centrifugation (17,000 × *g*, 4 °C, 10 min). The fresh cell extract (supernatant) was immediately used for enzyme activity measurements. Cell extracts for SDS-PAGE analysis or Bradford assays were stored at −20 °C.

### 4.5 Enzyme assays

All enzyme assays were performed aerobically in 0.5 ml reaction mixture either in glass cuvettes (spectrophotometric assays) or in Eppendorf vials (LC-MS assays). If not indicated otherwise, measurements were conducted at 28 °C. Cell extracts with protein concentrations > 1 mg ml^–1^ were used within 4 h after preparation (for isocitrate lyase activity measurements: 8 h). In cell extracts intended for LC-MS analysis, endogenous metabolites were depleted by ultrafiltration prior to the measurement (Vivaspin 20, 10 kDa MWCO, Sartorius), exchanging at least 90% of the initial solution by fresh resuspension buffer. Cell extract measurements for determination of specific activities were performed at least in biological duplicates, consisting of each ≥2 technical replicates for confirmation of protein dependency of the reaction; the number of replicates used in the individual experiments is indicated in the manuscript (Table 1). Assays for LC-MS analysis were performed in biological duplicates.

Acetone carboxylase was measured either spectrophotometrically at 365 nm as the acetone-dependent oxidation of NADH (NADH, ε_365nm_ = 3.4 mM^–1^ cm^–1^) by monitoring acetoacetate formation via a 3-hydroxybutyrate dehydrogenase-coupled assay (I) or qualitatively by detection of reaction products via LC-MS (II). The assay mixture contained 50 mM MOPS-KOH (pH 7.0), 10 mM MgCl_2_, 20 mM NH_4_Cl, 20 mM KHCO_3_, and cell extract. ITP (pH adjusted to ∼7.0) was added to final concentrations of 5 mM (assay I) or 2 mM (assay II). Besides, assay I contained 0.4 mM NADH and 0.02 mg of self-prepared 3-hydroxybutyrate dehydrogenase from *Rhodobacter sphaeroides* (HBDH; EC 1.1.1.30, Bergmeyer et al., 1967). HBDH catalyzes the NADH-dependent reduction of acetoacetate to 3-hydroxybutyrate. This reaction was monitored continuously. Reactions were started with 1 mM organic substrate or water in control experiments. For assay II, the reactions were stopped after 30 min (or 20 min for the measurements in Supplementary Figure 7) by mixing 100 μl of the sample with 100 μl of acetonitrile. Samples were centrifuged to remove the precipitated proteins and cell debris (17,000 × *g*, 4 °C, 10 min) and the supernatants were filtered through Minisart RC 4 filters (Sartorius). LC-MS measurements were performed within 4 days. Samples were kept frozen at −20 °C until measurement.

Acetophenone monooxygenase and phenol hydroxylase were measured spectrophotometrically at 365 nm as the acetophenone- or phenol-dependent oxidation of NADH or NADPH (NADPH, ε_365nm_ = 3.5 mM^–1^ cm^–1^). The assay mixture contained 50 mM Tris-HCl (pH 7.5), 5 mM DTE, 5 mM MgCl_2_, 0.4 mM NAD(P)H, and cell extract. Reactions were started with 1 mM acetophenone, 1 mM phenol, or water. For acetophenone monooxygenase measurements, all assays were performed under identical conditions once with water and once with acetophenone. Background activities with water were subtracted from the activity values obtained with acetophenone. As phenol hydroxylase activity was not detectable at 28 °C, these measurements were performed at 38 °C.

Isocitrate lyase was measured spectrophotometrically at 324 nm as the isocitrate-dependent formation of glyoxylate phenylhydrazine complexes (glyoxylate phenylhydrazone, ε_324nm_ = 15 mM^–1^ cm^–1^). The assay mixture contained 30 mM imidazole-HCl buffer (pH 7.3), 5 mM MgCl_2_, 1 mM EDTA-Na_2_, 4 mM phenylhydrazine, and cell extract. Reactions were started with 5 mM isocitrate.

### 4.6 Cloning

DNA purification, amplification, cloning, and transformation was performed using standard protocols (Ausubel et al., 2003). PCRs were routinely performed as gradient PCRs either with a self-prepared batch of Phusion polymerase or with Phusion polymerase from Thermo Fisher Scientific. Chemically competent *E. coli* DH5α cells were used for transformations.

The plasmid used for deletion of the *acrE* gene was constructed as follows: The *acrE* gene (NGP29002.1) was amplified from genomic *R. aetherivorans* DNA together with its flanking gene regions (791 bp upstream and 1017 bp downstream) using a forward primer (5′-TGACTG**TCTAGA**G ACCTCGACGAGGTATCCGGCGGTGTC-3′), which introduced an *Xba*I restriction site (bold), and a reverse primer (5′-TGAC TG**AAGCTT**GGACAGTTCGGCGGGTACGGCTGCAACAC-3′), which introduced a *Hind*III restriction site (bold). The following PCR conditions were used: Initial denaturation (98 °C, 60 s), 35 cycles of denaturation (98 °C, 30 s), primer annealing (temperature gradient from 55 °C to 65 °C, 30 s), and elongation (72 °C, 80 s), followed by a final elongation step (72 °C, 10 min). The amplified *acrE* gene was isolated via gel extraction and cloned into a pUC19 vector by restriction with *Xba*I and *Hind*III. The ligation mixture contained 30 ng vector and a 5-fold excess of insert and was incubated at 22 °C for 2 h, followed by transformation of the ligated plasmid. Positive clones were selected by blue-white screening. After purification and sequencing of the plasmid, the gene sequence was transferred to a pK19mobsacB suicide vector (Schäfer et al., 1994) by restriction with *Xba*I and *Hind*III. The ligation mixture contained 60 ng pK19mobsacB vector and a 5-fold excess of insert and was incubated at 22 °C for 2 h, followed by transformation of the ligated vector, purification, and sequencing (pK19mobsacB_NGP29002.1). A gentamicin resistance gene including promoter was amplified from a pBBR1MCS-5 plasmid (Kovach et al., 1995) using a forward primer (5′-TGACTG**AGTACT**GACGCACACCGTGGAAA-3′), which introduced a *Sca*I restriction site (bold), and a reverse primer (5′-CAGTCA**CACGATTGTG**GCGGCGTTGTGACAATTT-3′), which introduced an *Oli*I restriction site (bold). The PCR conditions were as follows: Initial denaturation (98 °C, 1 min), 35 cycles of denaturation (98 °C, 30 s), primer annealing (temperature gradient from 50 °C to 65 °C, 30 s), and elongation (72 °C, 30 s), followed by a final elongation step (72 °C, 10 min). The amplified gentamicin resistance gene was purified and cloned into the deletion vector by restriction with *Sca*I and *Oli*I. Restriction sites for both enzymes are naturally present at the edges of the *acrE* gene (*Sca*I, 205 bp downstream of the annotated *acrE* start codon; *Oli*I, 151 bp upstream of the *acrE* stop codon), enabling replacement of the gene. The ligation mixture contained 100 ng pK19mobsacB_NGP29002.1 and a 5-fold excess of the insert and was incubated at 22 °C for 15 h, followed by transformation of the ligated vector, purification, and sequencing.

The plasmid for complementation of the deletion mutants was constructed as follows: The *acrE* gene (NGP29002.1) was amplified from genomic *R. aetherivorans* DNA using a forward primer (5′-CA GTCA**GGTACC**GTGGTGATGGTGATGATGATGACCATCTC GTTTCCCGCCGTACTCGA-3′), which introduced a *Kpn*I restriction site (bold), and a reverse primer (5′-CAGTCA**GCGG** **CCGC**CGTTCAGGCACCGTGGCCGTCGAGCAGCC-3′), which introduced a *Not*I restriction site (bold). The following PCR conditions were used: Initial denaturation (98 °C, 60 s), 35 cycles of denaturation (98 °C, 30 s), primer annealing (temperature gradient from 55 °C to 70 °C, 30 s), and elongation (72 °C, 30 s), followed by a final elongation step (72 °C, 10 min). The pDD57 plasmid (DeLorenzo et al., 2018) was amplified by inverse PCR to remove the encoded *gfp* gene using a forward primer (5′-CAGTCA**G** **GTACC**ATGTATATCTCCTTCTTAATTAAGCATGCGGAT-3′), which introduced a *Kpn*I restriction site (bold), and a reverse primer (5′-CTGACT**GCGGCCGC**TGAATTCGAGCTGGATCCA GCTTCC-3′), which introduced a *Not*I restriction site (bold). The following PCR conditions were used: Initial denaturation (98 °C, 30 s), 35 cycles of denaturation (98 °C, 30 s), primer annealing (temperature gradient from 50 °C to 65 °C, 30 s), and elongation (72 °C, 2 min 10 s), followed by a final elongation step (72 °C, 10 min). Both amplified PCR products were isolated, restricted with *Not*I and *Kpn*I, and then ligated. The ligation mixture contained 100 ng inversely amplified pDD57 and a 3-fold excess of insert and was incubated at 22 °C for 2 h, followed by transformation of the ligated vector, purification, and sequencing.

### 4.7 Generation of deletion mutants

Pre-cultures of *E. coli* WM 3064 harboring the deletion plasmid and of *R. aetherivorans* were grown at 28 °C in LB medium for 1 day. Cultures were pelleted, weighed, and mixed in a 1:3 mass ratio (*E. coli*: *R. aetherivorans*). 50 μl of the cell mixture was applied as a single drop to an LB plate containing 0.1 mM DAP and incubated at 28 °C for 1–2 days. The grown colony material was streaked out on LB plates lacking DAP, but containing 20 μg ml^–1^ gentamicin. Plates were incubated at 28 °C. After 4 days, colony material was used as inoculum for liquid LB-gentamicin cultures. Grown cultures were passaged into culture media without antibiotics twice, then streaked out on LB agar containing 10% (w/v) saccharose, and incubated at 28 °C for 4 days. Single colonies were picked, transferred to liquid LB-gentamicin medium, and incubated at 28 °C for 3 days. Success in deletion of the *acrE* gene was verified by growing *R. aetherivorans* WT vs. Δ*acrE* deletion mutants on LB medium containing 10 mM acetone. Whole cell proteomics (each 3 biological replicates) with exponentially harvested cells (OD 0.2–0.4 when inoculating to a starting OD of 0.01) revealed that the gene products of the *acr* operon were strongly induced in both WT and Δ*acrE*, except for Acrε, which was detected only in WT cells (Supplementary Table 3). Furthermore, the genotype was verified by PCRs across the two flanking regions of the deleted gene. The upstream flank was verified using a forward primer (5′-GCAGGAGGTCATGGAGGAGGAGGTGC-3′) and a reverse primer (5′-CCATGAAAACCGCCACTGCGCCGTTACC-3′) with the following PCR conditions: Initial denaturation (98 °C, 30 s), 35 cycles of denaturation (98 °C, 30 s), primer annealing (temperature gradient from 55 °C to 65 °C, 30 s), and elongation (72 °C, 45 s), followed by a final elongation step (72 °C, 10 min). The downstream flank was verified using a forward primer (5′-GCTGCTCTTGATCTTTTCGGTCGTGAGTTCG-3′) and a reverse primer (5′-GCTCCTCGGATTCCTCCATGGTGGC-3′) with the following PCR conditions: Initial denaturation (98 °C, 30 s), 35 cycles of denaturation (98 °C, 30 s), primer annealing (temperature gradient from 50 °C to 65 °C, 30 s), and elongation (72 °C, 2 min), followed by a final elongation step (72 °C, 10 min). The PCR products had the correct size and their identity was additionally confirmed by sequencing (Supplementary Figure 8 and Supplementary Table 8). The *acrE* deletion mutant was cryo-conserved at −80 °C in LB medium containing 25% glycerol. Transformation of the deletion mutants with a pDD57 plasmid carrying a cloned *acrE* gene was tested by electroporation and conjugation. Cells acquired kanamycin resistance only with the latter method, indicating successful plasmid uptake. However, when transferring cells from LB medium to minimal medium containing 10 mM acetone and 0–50 μg ml^–1^ kanamycin, growth was not restored.

### 4.8 Preparation and transformation of electrocompetent *R. aetherivorans* cells

Electrocompetent *R. aetherivorans* cells were prepared as described previously (Ngo et al., 2025). 5 ml LB tubes were inoculated with colony material from LB agar plates and incubated at 28 °C overnight. Then, 100 ml of LB medium were inoculated to a starting OD of 0.05 and incubated in an Erlenmeyer flask shaking at 130 rpm at 28 °C. To destabilize the cell envelope and improve plasmid uptake, 1.5% glycine was added to the medium aseptically after autoclaving. Cells were grown to an OD of 0.57 and then harvested by centrifugation (2,370 × *g*, 4 °C, 30 min.). The supernatant was discarded and the cells were washed with ice-cold deionized water twice. Then, cells were resuspended to an OD of 3.7 in 10% (v/v) glycerol, frozen in liquid nitrogen, and stored at −80 °C.

Electrocompetent cells (100 μl aliquots) were thawed on ice for 30 min. Then, 1.5 μl of plasmid solution (32.5 ng μl^–1^) were added to the cells. The solution was mixed by pipetting, transferred to an electroporation cuvette (PeqLab, #71-2010, 1 mm gap), and incubated on ice for 30 min. Electroporation was performed with a MicroPulser electroporator (Bio-Rad, 1.8 kV, 1 pulse). Immediately after application of the pulse, cells were suspended in 900 μl of SOC medium (20 g l^–1^ tryptone, 5 g l^–1^ yeast extract, 8.55 mM NaCl, 2.5 mM KCl; added after autoclaving: 10 mM MgCl_2_, 20 mM glucose; pH 7.0). The cell suspension was incubated aerobically at 28 °C for 4 h, streaked out on LB agar plates containing 50 μg ml^–1^ kanamycin, and then incubated at 28 °C.

### 4.9 Whole cell proteomics

Whole cell proteomics were conducted in biological triplicates. Frozen cell pellets of *R. aetherivorans* were resuspended in lysis buffer (20 mM Tris-HCl, pH 7.5, 50 mM NaCl, 5 mM EDTA). Fresh stock solutions of protease inhibitor cocktail (Roche) and lysozyme (Sigma-Aldrich) were added to the resuspended cell pellets to final lysozyme concentrations of 0.5 g l^–1^. The resuspended cell pellets were incubated at 30 °C for 30 min. Then, sodium lauroyl sarcosinate (SLS) was added to the solutions to a final concentration of 2% and the samples were incubated at 90 °C for 60 min, followed by a 60 s ultrasonic treatment with a Vial Tweeter (Hielscher). After determination of protein concentrations, the dissolved proteins were reduced by incubation with 5 mM tris(2-carboxyethyl)phosphine (TCEP) at 90 °C for 10 min and subsequently alkylated with 10 mM iodoacetamide (25 °C, 30 min). Aliquots of each 50 μg protein were rebuffered using SP3 Protein Clean-up according to the manufacturer’s instructions and digested overnight with 1 μg trypsin (SERVA) in 150 μl of 10% acetonitrile/50 mM ammonium bicarbonate. The peptide solutions were purified using C18 Chromabond Microspin columns (Macherey-Nagel). The solutions were applied to the equilibrated cartridges, washed with 0.1% trifluoroacetic acid (TFA), and eluted with 50% acetonitrile and 0.1% TFA. Then, the peptides were dried in a SpeedVac and reconstituted in 0.1% TFA prior to liquid chromatography-mass spectrometry (LC-MS) analysis.

Liquid chromatography-mass spectrometry analysis was carried out on an Orbitrap Exploris 480 instrument connected to an Ultimate 3000 RSLC nano and a nanospray flex ion source (all from Thermo Fisher Scientific). Peptide separation was performed on a reverse phase HPLC column (75 μm ×42 cm) packed in-house with C18 resin (2.4 μm; Dr. Maisch). The following separating gradient was used: 94% solvent A (0.15% formic acid) and 6% solvent B (99.85% acetonitrile, 0.15% formic acid) to 25% solvent B over 40 min, and an additional increase of solvent B to 35% for 20 min at a flow rate of 300 nl min^–1^. Peptides were ionized at a spray voltage of 2.3 kV. 445.12003 m/z was used as an internal calibrant. The funnel radio-frequency (RF) level was at 40, and heated capillary temperature was at 275 °C.

Mass spectrometry raw data were acquired on an Exploris 480 instrument (Thermo Fisher Scientific) in data-independent acquisition mode (DIA). For DIA experiments, full MS resolutions were set to 120.000 at m/z 200 and the full MS, AGC (Automatic Gain Control) target was 300% with an injection time (IT) of 50 ms. AGC target value for fragment spectra was set at 3000%. 45 windows of 14 Da were used with an overlap of 1 Da from m/z 349.5 to 980.5. Resolution was set to 15,000 and IT to 22 ms. Stepped higher-energy collisional dissociation (HCD) collision energy of 25%, 27.5%, and 30% was used. MS1 data were acquired in profile, and MS2 DIA data in centroid mode.

Analysis of DIA data was performed using DIA-NN version 1.8 against a UniProt protein database from *R. aetherivorans* (12 June 2024; 5,858 protein entries). Searches allowed up to two missed cleavages, with methionine oxidation as a variable modification and cysteine carbamidomethylation as a fixed modification. Match-between-runs, interference removal, smart profiling, and retention time (RT)-dependent normalization were enabled. Protein inference was gene-based, precursor false discovery rate (FDR) was set to 1%, and downstream analysis was performed using a modified SafeQuant script for DIA-NN outputs. To assess the statistical significance of differential protein abundance between two experimental conditions, *p*-values were calculated using Welch’s *t*-tests.

### 4.10 Mass spectrometric protein identification from SDS gel bands

The gel bands were destained and rebuffered in a mixture of 50% acetonitrile, 60 mM (NH_4_)_2_CO_3_, and 30 mM thioglycolic acid (pH 8.2) and then dried. Subsequently, they were digested with 0.005 g l^–1^ trypsin (Serva) in a solution of 10% acetonitrile and 50 mM NH_4_HCO_3_ overnight. The resulting peptide solution was treated with 0.2% TFA and 80% acetonitrile and vortexed (1,200 rpm, 30 °C, 10 min). After transferring the supernatant to new tubes, this step was repeated. Then, extracts were vacuum dried, acidified with 0.1% TFA, vortexed (1,000 rpm, 30 °C, 5 min), purified using C18-SPE Microspin columns as described above, and then subjected to liquid chromatography-mass spectrometry (LC-MS). LC-MS analysis was carried out as described above, but using a data-dependent acquisition (DDA) method and a separation gradient of 0.15% formic acid/acetonitrile for 30 min. The data acquisition mode was set to include a high-resolution MS scan with a resolution of 60,000 half-widths, followed by MS/MS scans of the most intense ions. The MS data were interpreted with Proteome Discoverer 1.4 and the *R. aetherivorans* protein database downloaded from UniProt. The exact search parameters are specified in Supplementary Table 9.

### 4.11 Estimation of the abundance of acetone carboxylase in cell extract

The abundance of acetone carboxylase was estimated in four biological replicates based on the intensity of SDS gel bands containing the three large Acr subunits (Supplementary Figure 5) using the software ImageJ (Schneider et al., 2012). The gel image was converted to grayscale and its tones were inverted (i.e., light bands on dark background). Absolute background intensities on the gel were determined for ten different patches between the lanes (integrated density measurement) and the average background intensity per units of area was calculated. For each cell extract, absolute intensities of either the entire lane or of the two bands containing the three large Acr subunits were measured and corrected by subtracting the average background intensities normalized to the respective areas. To calculate the proportion of Acr in the cell extract, the intensities of both Acr bands were summed up and divided by the intensity of the respective entire lanes. As bands corresponding to subunits Acrβ and Acrε were not included in the analysis, the actual Acr proportions are slightly higher than the calculated values.

### 4.12 Metabolomics

Qualitative determination of acetoacetate, 3-oxopentanoate, 3-oxohexanoate, 4-oxohexanoate, 3-oxoheptanoate, and benzoylacetate was performed using high-resolution electrospray ionization (HRES) LC-MS. Chromatographic separation was conducted at 25 °C on an Agilent Infinity II 1290 HPLC system using a Kinetex EVO C18 column (150 × 2.1 mm, 3 μm particle size, 100 Å pore size, Phenomenex) connected with a guard column of similar specificity (20 × 2.1 mm, 3 μm particle size, Phenomenex), applying a constant flow rate of 0.2 ml min^–1^. The mobile phases consisted of 0.1% formic acid either in water (phase A) or in methanol (phase B, Honeywell). After injection of 5 μl sample, the following protocol was used: Constant flow with 0% phase B (4 min), linear gradient from 0% to 100% phase B (2 min), constant flow with 100% phase B (1 min), linear gradient from 100% to 0% phase B (0.1 min), and constant flow with 0% phase B (4.9 min).

Except for the measurements in Supplementary Figure 1, an Agilent 6546 Q-TOF mass spectrometer was used in negative ionization mode with a dual electrospray ionization source. The following conditions were applied: Electrospray ionization (ESI) spray voltage 2000 V, nozzle voltage 500 V, sheath gas 300 °C at 11 l min^–1^, nebulizer pressure 45 psig, and drying gas 170 °C at 5 l min^–1^. The instrument was used in low molecular weight mode with a screening window of 50–1700 m/z and a scan rate of 1.5 spectra s^–1^. 112.9856 and 1033.9881 were used for online mass calibration. Compounds were identified based on their accurate mass within a mass window of 5 ppm. For acetoacetate, retention times were compared to a standard. Chromatograms were integrated using MassHunter software (Agilent).

For the measurements in Supplementary Figure 1, an Agilent 6495 ion funnel QQQ mass spectrometer was used for acetoacetate determination. The device was operated in negative mode with an electrospray ionization source, applying the following conditions: ESI spray voltage 2000 V, nozzle voltage 500 V, sheath gas 300 °C at 11 l min^–1^, nebulizer pressure 50 psig, and drying gas 80 °C at 16 l min^–1^. Acetoacetate was identified based on its mass transition and retention time compared to a standard. Again, chromatograms were integrated using MassHunter software (Agilent). Mass transitions, collision energies, Cell accelerator voltages, and Dwell times were optimized using a chemically pure acetoacetate standard. Parameter settings are given in Supplementary Table 10. For LC-MS raw data, see Supplementary Table 11.

### 4.13 Phylogenetic analysis

The protein sequence of *Rhodococcus aetherivorans* Acrβ (*acrB* gene product, NGP29005.1) was used as query to search for homologs in NCBI-refseq_select using the BLASTP online tool^1^. The expect threshold was set to 1e-5. Uncultured and environmental samples were excluded. This initial search yielded 503 results. All hits were checked manually for their presence within an *apc*-like operon containing additional genes for three hydantoinase homologs and one putative subunit ε. For sequences which appeared in several genomes (i.e., BLAST MULTISPECIES annotations), operon conservation was checked only once unless the species were discussed more in detail in the section “2 Results” part. All hits for which the *acrB* gene was not present in an *apc*-like operon or for which the neighboring genes were not known (e.g., gene at the end of a contig) were removed as false positive. Highly fragmented operons were removed, too. Note that in some cases the operons were interrupted by one (conserved) additional gene, in many cases annotated as glucose 1-dehydrogenase; these operons were highlighted in Supplementary Table 5, but still included in the analysis. Moreover, all operons with (partly) incomplete protein sequences were removed. At expect thresholds of ≥3e-15, the majority of hits turned out to be false positive. Hence, all sequences with expect thresholds of ≥3e-15 were excluded. After these filtering steps, 219 sequences were left (Supplementary Table 5). The protein sequences of either Acrβ (*acrB*) or putative Acrε (*acrE*) subunits were aligned using MUSCLE (Edgar, 2004) implemented in MEGA12 (Kumar et al., 2024) using default parameters (cluster method: UPGMA). Phylogenetic trees were constructed using the Maximum Likelihood algorithm, again in MEGA12 (Kumar et al., 2024) using default parameters with the Jones-Taylor-Thornton model (Jones et al., 1992). In addition, bootstrapping was performed in MEGA12 with 500 bootstrap replicates. iTOL (Letunic and Bork, 2007) was used for visualization. When evaluating the distribution of *acr* operons across genomes of *Rhodococcus* species, genomes which failed the NCBI completeness check were excluded from the analysis. In consequence, all analyzed genomes had a completeness of >94% (NCBI CheckM values).

### 4.14 Creation of Hidden Markov models (HMMs)

For the creation of Hidden Markov models, either the Acrε subunit from *R. aetherivorans* (NGP29002.1) or the Apcε subunit from *A. aromaticum* (WP_011237166.1) were used as seed sequences to search against the NCBI-nr database (see Footnote 1). The NCBI-nr database was used to identify the largest number of different protein sequences (compared to NCBI-refseq used previously for construction of the phylogenetic trees), hence improving the respective Hidden Markov models. The expect thresholds were set to 1e-5 and the searches yielded 867 hits with *R. aetherivorans* or 116 hits with *A. aromaticum* subunit ε proteins. The respective sequences were aligned using MUSCLE (Edgar, 2004) implemented in MEGA12 (Kumar et al., 2024) using default parameters (cluster method: UPGMA). Alignments were loaded into AliView (Larsson, 2014) and manually edge-trimmed to remove low occupancy positions. Incomplete sequences were excluded (*R. aetherivorans*, 41 sequences; *A. aromaticum*, 6 sequences). The Hidden Markov models were built using hmmbuild and hmmsearch was used to screen all sequences from the phylogenetic tree of putative subunits ε for homology (Eddy, 2009). The results of HMM searches are provided in Supplementary Tables 6, 7.

### 4.15 Prediction and comparison of protein structures

The three-dimensional structures of Acrε from *R. aetherivorans* and Apcε from *A. aromaticum* were predicted using AlphaFold3 with default settings (Abramson et al., 2024) on the AlphaFold Protein Structure Prediction Server^2^. The full-length amino acid sequences were submitted (Acrε, NGP29002.1; Apcε, WP_011237166.1). After prediction, the structures were aligned and visualized using PyMOL (The PyMOL Molecular Graphics System, Version 3.1.8 Schrödinger, LLC). Foldseek was used for comparison of the structures (van Kempen et al., 2024).

### 4.16 Calculation of specific *in vivo* substrate consumption rates of *R. aetherivorans*

Specific growth rates (μ) were calculated as ln2 divided by the culture’s doubling times (acetone, 0.24 h^–1^; acetophenone, 0.11 h^–1^). Specific substrate consumptions (dS) per time unit (dt) were calculated as described previously (Hügler et al., 2003) using the following formula: dS/dt = (μ/Y)⋅X, with Y representing the growth yield (0.5 g of dry mass formed per 1 g carbon fixed) and X representing the cell dry mass in g (1 g of dry mass corresponding to 0.5 g of protein). For acetone, we assumed co-assimilation of stoichiometric amounts of HCO_3_^–^. For the calculation of acetophenone consumption, a correction factor of 1.6 was used because the carbon portion in acetophenone is 80% rather than the cellular 50%.

### 4.17 Other methods

SDS-PAGE (10% or 7% acrylamide) was performed according to Laemmli (1970). In most cases, protein concentrations were determined with Bradford assays using bovine serum albumin (BSA) as a standard (Bradford, 1976). During sample preparation for whole cell proteomics, protein concentrations were measured using a BCA protein assay kit (Thermo Fisher Scientific).

## Data Availability

The original contributions presented in this study are included in this article/Supplementary material, further inquiries can be directed to the corresponding author.

## Footnotes

1
https://blast.ncbi.nlm.nih.gov/

2
https://alphafoldserver.com/
