# Supplementary material for: An acetophenone carboxylase homolog from Rhodococcus aetherivorans initiates acetone rather than acetophenone catabolism
Source: Front Microbiol. 2026 Oct 5;17:1888828. doi: 10.3389/fmicb.2026.1888828 (PMC13677872; doi:10.3389/fmicb.2026.1888828)
Supplement: Supplementary file 1 [file Supplementary_file_1.zip › Supplementary Material.pdf]

Supplementary Material

Supplementary Figures

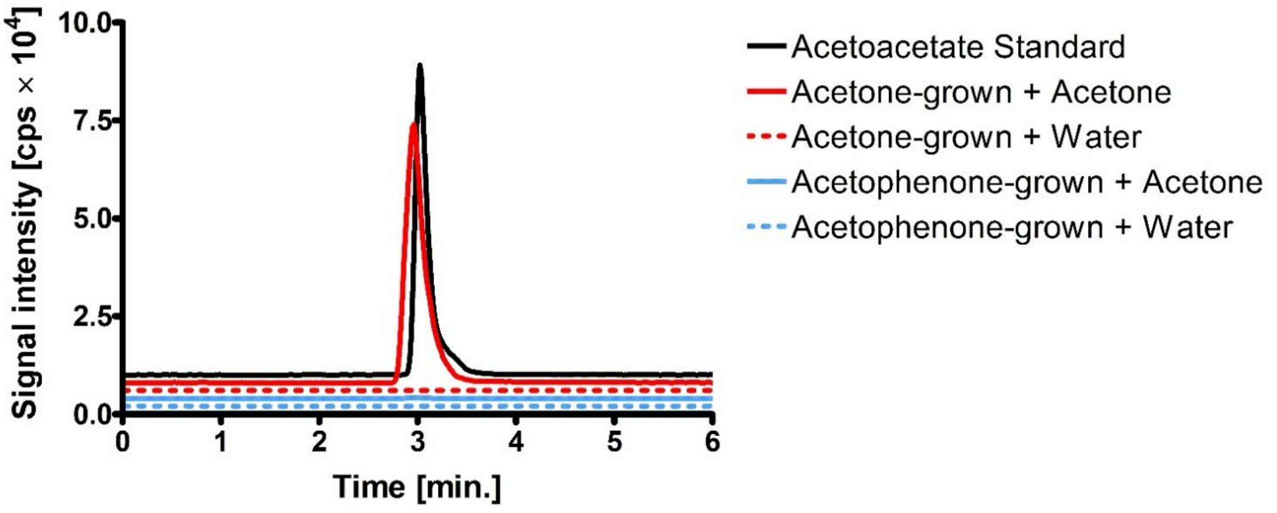

Supplementary Figure 1: LC-MS-based detection of acetoacetate produced in acetone carboxylase assays with extracts from acetone- or acetophenone-grown cells. The diagram shows an overlay of the extracted chromatograms of acetoacetate.

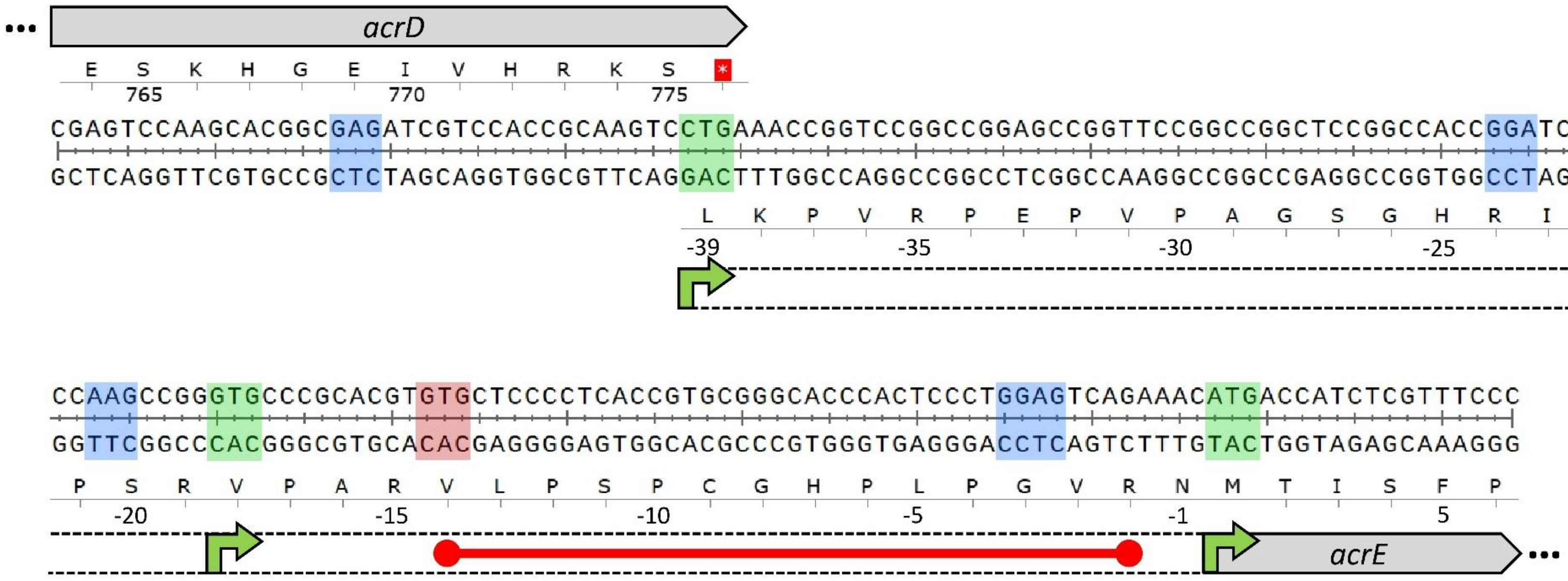

Supplementary Figure 2: Putative N-terminal extension of the annotated open reading frame of Acrε. The displayed sequence ranges from the end of the annotated *acrD* gene to the beginning of the annotated *acrE* gene. Mass spectrometric analyses revealed the presence of a fragment (red line) encoded upstream of the annotated *acrE* start codon, suggesting the use of an alternative start codon. The annotated ATG start codon and putative alternative CTG or GTG codons are highlighted in green and labelled with green arrows. Putative Shine-Dalgarno sequences are shown in blue. Based on the sequence of the identified peptide fragment, we exclude that the GTG codon at the -14 position (highlighted in red) serves as an alternative start codon.

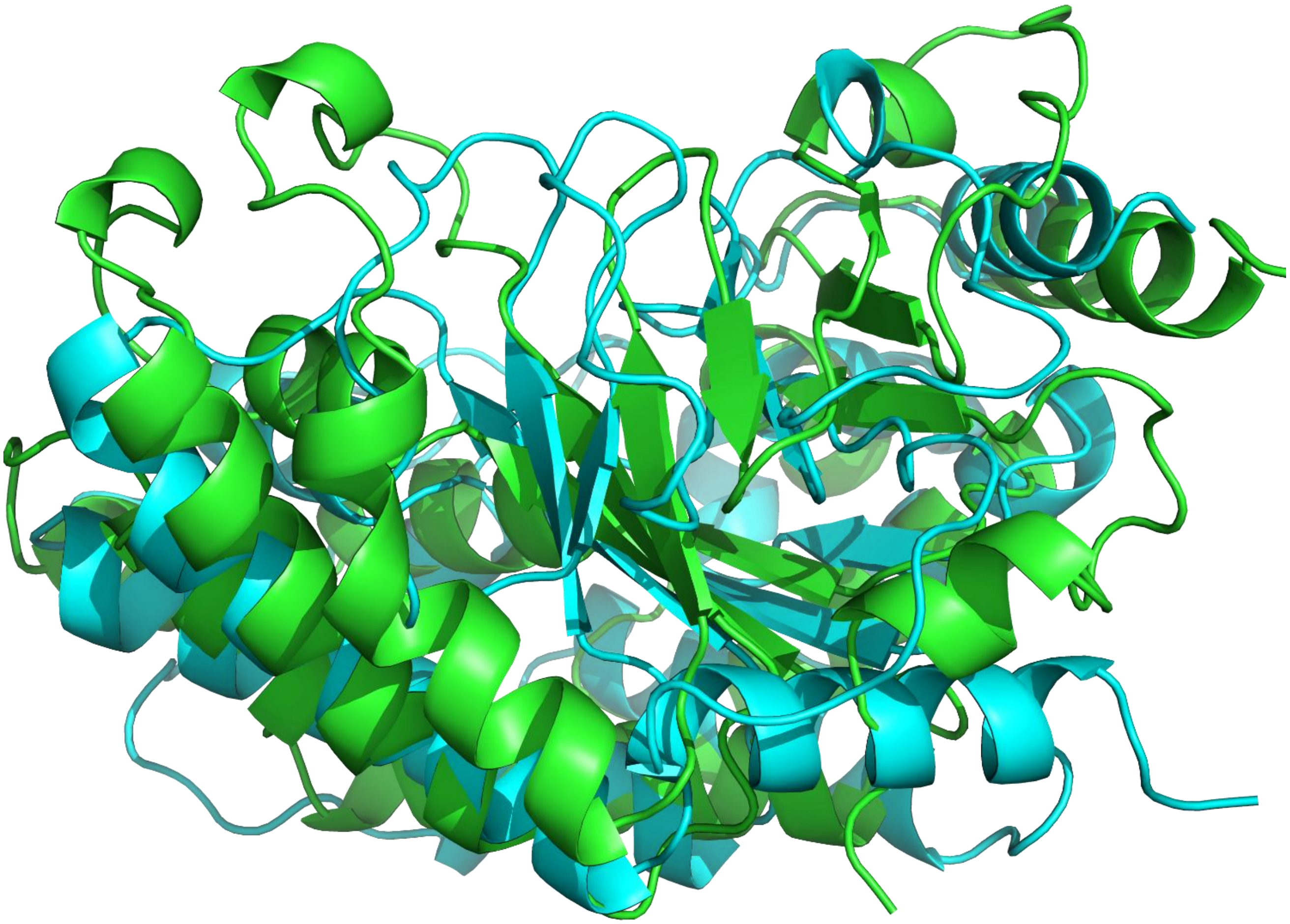

Supplementary Figure 3: Structural alignment of AlphaFold models of Acrε from *R. aetherivorans* and Apcε from *A. aromaticum*. AlphaFold models were aligned with PyMOL and the overall RMSD value was 11.671. Acrε from *R. aetherivorans* (NGP29002.1) is shown in green and Apcε from *A. aromaticum* (WP_011237166.1) in cyan.

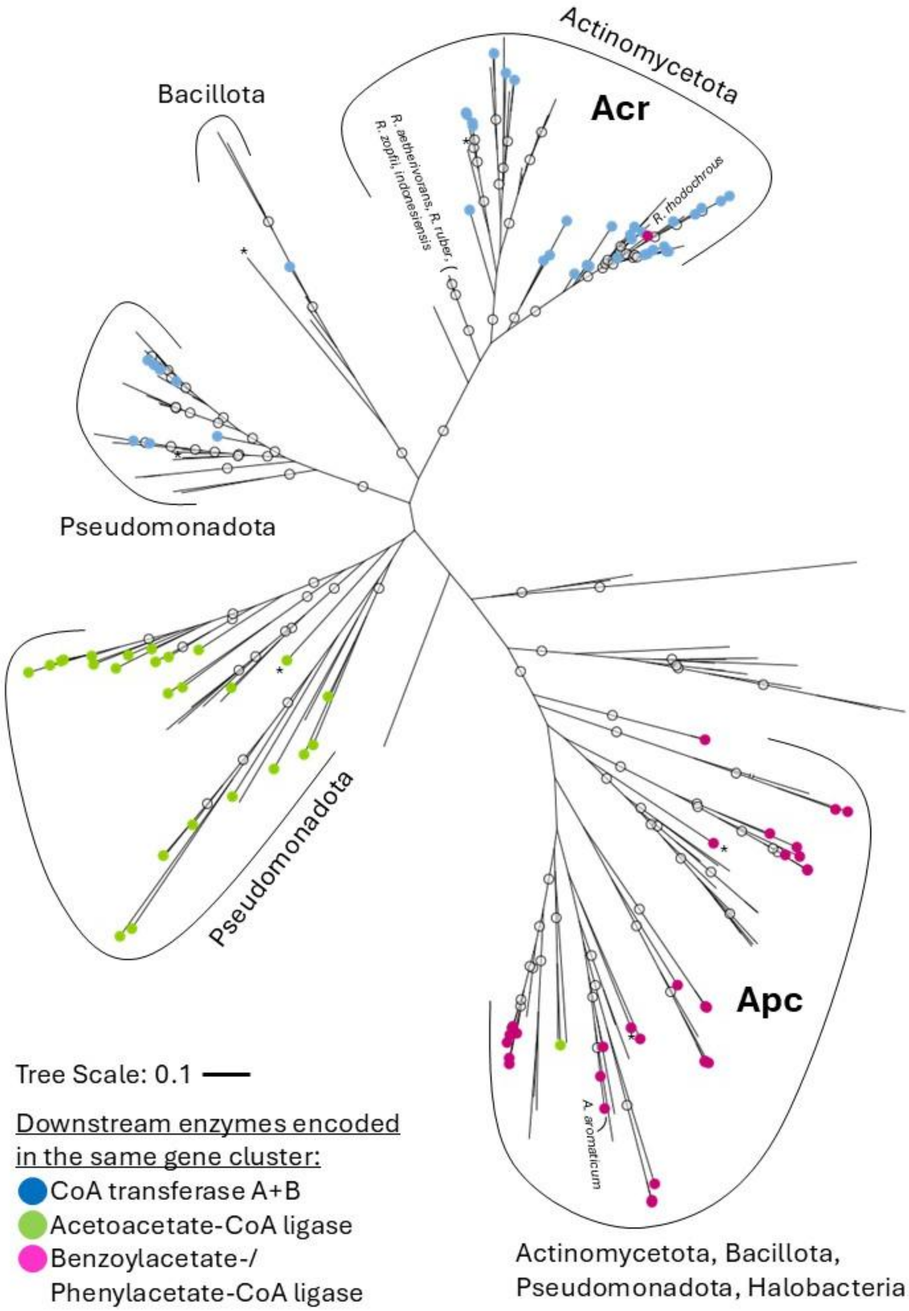

Supplementary Figure 4: Phylogenetic tree of Apcβ/Acxγ homologs from heterodecameric ketone carboxylases. Apcβ/Acxγ subunits were identified (Suppl. Table 5), the corresponding genes were screened for their presence within an *apc*-like operon (*i.e.*, encoding three hydantoinase/oxoprolinase homologs, one Apcβ/Acxγ homolog, and one additional hypothetical protein of ca. 30 kDa), and the protein sequences of the Apcβ/Acxγ homologs were aligned via MUSCLE (Edgar, 2004). The phylogenetic tree was constructed using the Maximum Likelihood algorithm with the Jones-Taylor-Thornton model (Jones et al., 1992) implemented in MEGA12 (Kumar et al., 2024). Bootstrapping was performed in MEGA12 with 500 bootstrap replicates. Bootstrap values ≥ 0.7 are displayed in the figure. iTOL (Letunic and Bork, 2007) was used for visualization. The scale bar represents 0.1 amino acid substitutions per site. Marked with an asterisk (*): Other phylogenetic classification than indicated.

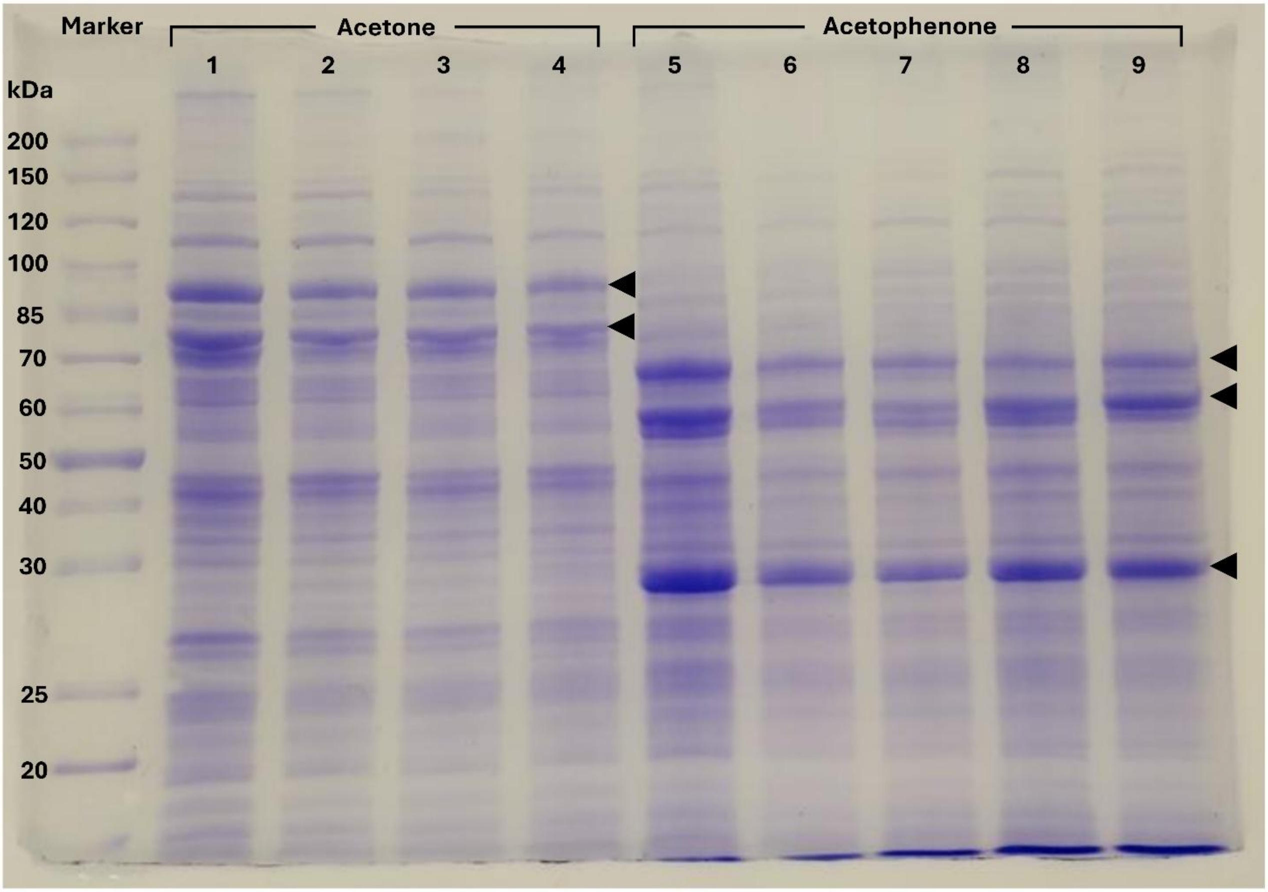

Supplementary Figure 5: SDS-PAGE of various *R. aetherivorans* extracts from acetone- and acetophenone-grown cells. 20 µg protein were loaded per lane. The arrows indicate the position of acetone carboxylase subunits in acetone-grown cells and oxygenases in acetophenone-grown cells.

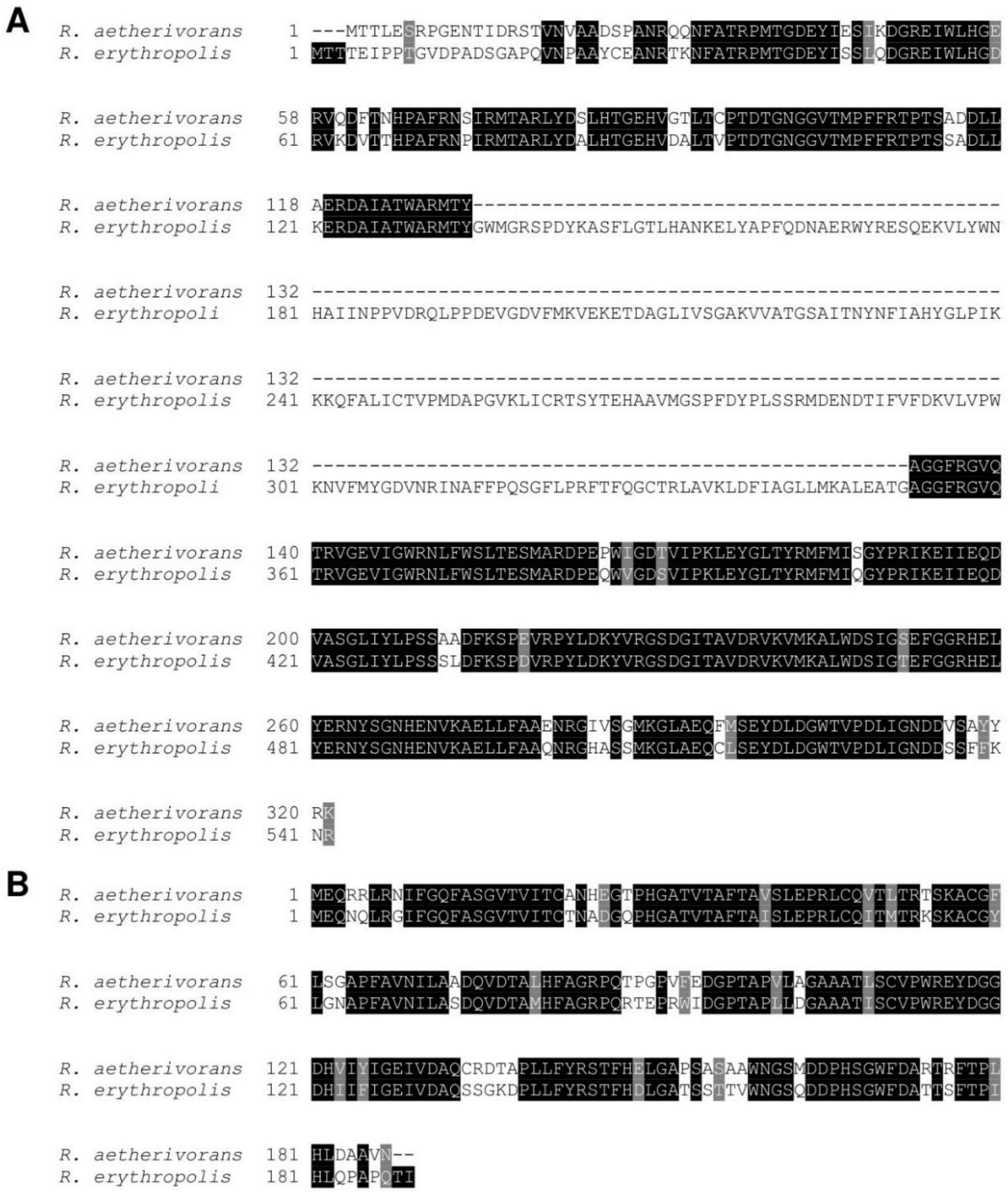

Supplementary Figure 6: Alignment of the *R. aetherivorans* phenol hydroxylase candidate subunits with the subunits of an experimentally confirmed phenol hydroxylase from *R. erythropolis*. (A) Alignment of phenol hydroxylase subunit PheA1 from *R. erythropolis* (ABS30825.1) with WP_277606666.1 (NCBI locus tag: G6039_RS13570) and WP_277606685.1 (NCBI locus tag: G6039_RS26435) from *R. aetherivorans* DSM 44752. **(B)** Alignment of phenol hydroxylase subunit PheA2 from *R. erythropolis* (ABS30826.1) with WP_043802490.1 (NCBI locus tag: G6039_RS13575) from *R. aetherivorans* DSM 44752. Alignments were performed using ClustalW (Thompson et al., 1994) and visualized using the Boxshade online tool (https://junli.netlify.app/apps/boxshade/). Sequence identity is indicated by black background; sequence similarity is indicated by grey background. Experimental evidence for phenol hydroxylase activity of the *R. erythropolis* enzyme is provided in Saa et al. (2010).

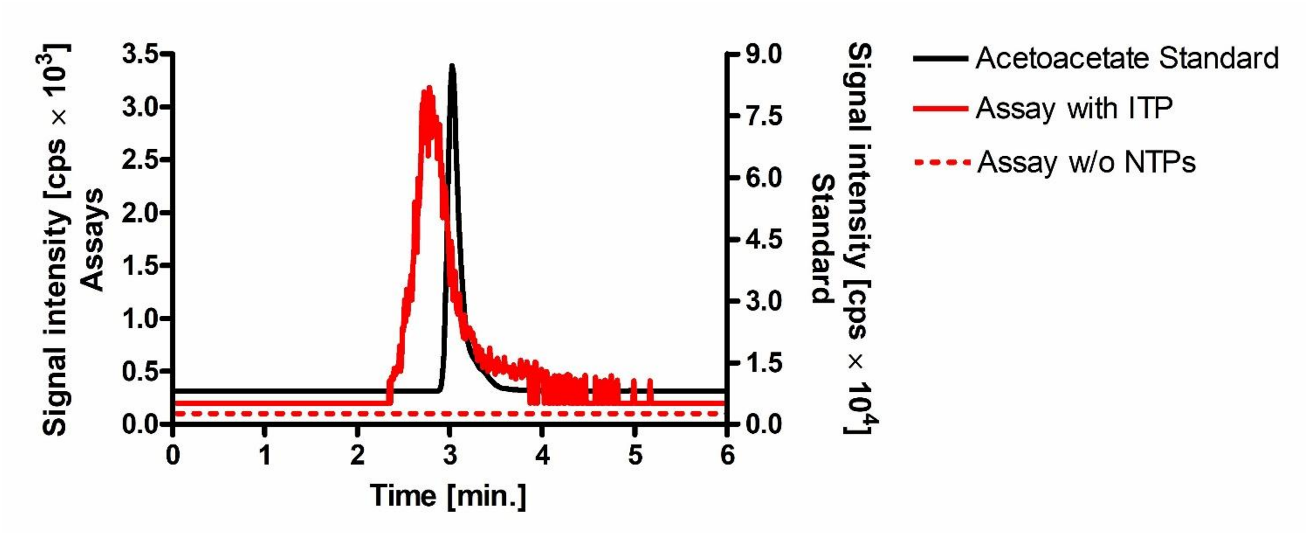

Supplementary Figure 7: LC-MS-based confirmation of the nucleotide dependency of the acetone carboxylase reaction in extracts from acetone-grown cells. Assays were performed either with ITP or in the absence of any nucleotide triphosphates (NTPs) and acetoacetate formation was measured by LC-MS. The diagram shows an overlay of the extracted chromatograms of acetoacetate. The peak identity has been confirmed by mass determination and the deviation in elution time between sample and standard is probably due to slight differences in the sample matrix. Additional negative controls with heat-inactivated cell extracts or water replacing the substrate did not result in acetoacetate production and are not shown here.

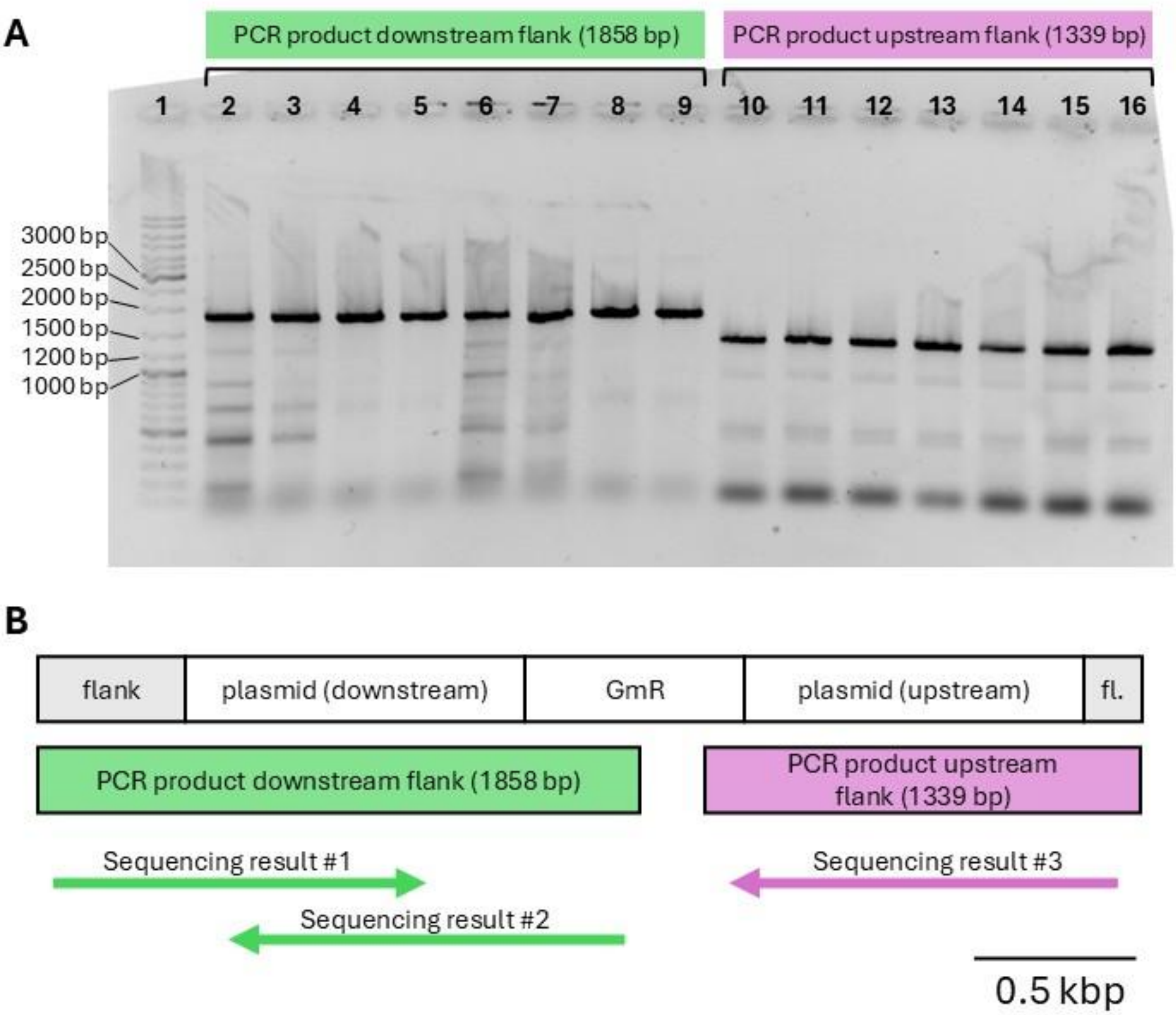

Supplementary Figure 8: Confirmation of correct *acrE* deletion by PCRs across the modified gene locus. (A) Gel electrophoresis and **(B)** Scheme of the respective PCR products. Each one of the primers bound within the introduced gentamicin gene, while each a second primer bound in the genomic regions up- or downstream from the sequence encoded on the deletion plasmid. The sequencing results are provided in Supplementary Table 8.

Supplementary Tables

Please note that **Supplementary Tables 2, 3, 6, 7, and 11** are provided as separate files.

Supplementary Table 1: Mass spectrometric protein analysis of SDS-PAGE bands from the gel shown in Fig. 1C. Raw data searches were performed against *Rhodococcus aetherivorans* proteins in the UniProt database (for exact search parameters, see Suppl. Table 9). For every gel band, hits were sorted by PSM values and the top hit(s) were displayed in the table. Note that the two smaller subunits of acetone carboxylase (Acrβ and Acrε) were not visible on the gel and hence could not be analyzed here.

Gel Band	UniProt Accession	NCBI Protein Accession	Current Annotation **(proposed actual function in red)**	Molecular Weight [kDa]	Score	Coverage	Peptides	PSM
**D**	A0A059MLZ3	NGP29003.1	Hydantoinase B/oxoprolinase family protein **(AcrD)**	86.3	935.18	42.84%	57	1760
**C+A**	N1MBT0	NGP29004.1	Hydantoinase B/oxoprolinase family protein **(AcrC)**	77.1	999.39	45.06%	36	831
**C+A**	A0AA46P6L6	NGP29006.1	Hydantoinase B/oxoprolinase family protein **(AcrA)**	67.5	715.30	43.97%	39	648
**16**	N1MA34	WP_006937797.1	NAD(P)/FAD-dependent oxidoreductase, Flavin-containing monooxygenase, Cyclohexanone monooxygenase, Putative **acetophenone monooxygenase (?)**	61.1	338.39	57.19%	56	481
**16**	A0A059MK19	WP_006932131.1	60 kDa chaperonin (GroEL)	58.1	195.28	59.38%	50	276
**3**	A0AA46PWI7	WP_263508839.1 (WP_277606666.1 + WP_277606685.1)	Pyoverdin chromophore biosynthetic protein pvcC, 4-Hydroxyphenylacetate 3-hydroxylase family protein, Putative **phenol monooxygenase**, large subunit *(*Note: From* R. aetherivorans *N1; Homolog also present in* R. aetherivorans *DSM 44752, but split between two contigs)*	60.4	132.23	17.25%	11	212
**3**	A0AA46PAG4	WP_029546750.1	Catalase *(*Note: From* R. aetherivorans *N1)*	54.2	81.59	19.59%	12	210
**5**	A0AA46P5E0	WP_029546511.1	Catechol 1,2-dioxygenase	31.6	24.34	3.18%	1	29

Supplementary Table 4: Substrate-independent, uncoupled NADPH oxidation in acetophenone-grown cells. The mean value of specific acivity ratios (acetophenone/water) is 2.62 ± 0.43 (n = 5).

**Biological replicate**	**Measurement**	**% Cell extract in the assay mixture**	**Dilution factor**	**Substrate (start of the reaction)**	**Specific activity [mU/mg]**				**Specific activity ratio (Acetophenone/Water)**
					Measured	Background-corrected	Mean	STD	
**#1**	1	20	5	Acetophenone	11.0	7.05	7.50	0.63	2.77
	2	20	5	Water	3.99				
	3	10	10	Acetophenone	14.5	7.94			2.22
	4	10	10	Water	6.51				
**#2**	1	10	10	Water	6.30	9.07	9.30	0.32	2.44
	2	10	10	Acetophenone	15.4				
	3	20	5	Water	4.80	9.52			2.98
	4	20	5	Acetophenone	14.3				
**#3**	1	10	10	Acetophenone	7.06	4.03	3.49	0.70	2.33
	2	10	10	Water	3.03				
	3	20	5	Water	1.43	2.70			2.89
	4	20	5	Acetophenone	4.13				
	5	15	6.67	Water	1.98	3.73			2.88
	6	15	6.67	Acetophenone	5.71				
**#4**	1	10	10	Acetophenone	12.08	7.72	6.18	1.74	2.77
	2	10	10	Water	4.36				
	3	20	5	Water	4.07	4.29			2.05
	4	20	5	Acetophenone	8.36				
	5	15	6.67	Acetophenone	10.9	6.52			2.50
	6	15	6.67	Water	4.34				
**#5**	1	10	10	Acetophenone	24.8	17.74	15.0	3.85	3.50
	2	10	10	Water	7.09				
	3	5	20	Water	11.7	12.30			2.05
	4	5	20	Acetophenone	24.0				

Supplementary Table 5: Occurrence of gene clusters encoding for heterodecameric ketone carboxylases in different bacteria. The gene clusters were identified via a BLAST search for Apcβ/Acxγ homologs and additionally encoded for three hydantoinase homologs and a putative subunit ε, which was annotated as hypothetical protein in most cases. Where indicated, the operons were fragmented, *i.e.* the gene encoding the subunit ε was separated from the other four *acr* genes by a gene encoding for an additional protein with annotated function (*e.g.*, glucose 1-dehydrogenase). The gene clusters often contained genes encoding for putative acetoacetate- or benzoylacetate-converting enzymes, which is indicated in the table. In some cases, the downstream metabolic enzymes were not encoded within the *acr* operon, but only in close proximity (enzymes in parantheses).

**Acrβ NCBI Accession** *(MULTISPECIES entries in blue)*	**Strain** *(Note: For MULTISPECIES Accessions, only one representative strain is listed)*	**Phylogenetic group**	**Up- or downstream gene encoding for a putative acetoacetate- or benzoylacetate-converting enzyme?**	**Operon fragmented?**
**WP_006945515.1**	***Rhodococcus aetherivorans*** DSM 44752	Actinomycetota, *Nocardiaceae*		
**WP_017681267.1**	***Rhodococcus ruber*** PCTR3 13	Actinomycetota, *Nocardiaceae*		
**WP_010593155.1**	***Rhodococcus indonesiensis*** NPDC001044	Actinomycetota, *Nocardiaceae*		
WP_072809979.1	***Rhodococcus zopfii*** NPDC003403	Actinomycetota, *Nocardiaceae*		
WP_028931537.1	*Pseudonocardia asaccharolytica* DSM 44247	Actinomycetota, *Pseudonocardiaceae*	CoA transferase A+B	
**WP_386339722.1**	*Streptomyces xiangluensis*	Actinomycetota, *Streptomycetaceae*	CoA transferase A+B	
**WP_197651765.1**	*Carbonactinospora thermoautotrophica* StC	Actinomycetota, *Carbonactinosporaceae*	CoA transferase A+B	
WP_183099686.1	*Nocardioides pelophilus* CGMCC 4.7388	Actinomycetota, *Nocardioidaceae*	CoA transferase A+B	
WP_110502376.1	*Arthrobacter livingstonensis*	Actinomycetota, *Micrococcaceae*	CoA transferase A+B	
WP_176636431.1	*Arthrobacter wenxiniae* AETb3-4 38	Actinomycetota, *Micrococcaceae*	CoA transferase A+B	
**WP_381085119.1**	*Streptomyces caeni* CGMCC 4.7426	Actinomycetota, *Streptomycetaceae*	CoA transferase A+B	
WP_245542798.1	*Pseudoclavibacter soli*	Actinomycetota, *Microbacteriaceae*		
WP_234753558.1	*Arthrobacter ramosus* JCM 1334	Actinomycetota, *Micrococcaceae*		
**WP_006900985.1**	***Rhodococcus rhodochrous*** GD02	Actinomycetota, *Nocardiaceae*	CoA transferase A+B	
WP_040862464.1	*Nocardia niigatensis* NPDC003476	Actinomycetota, *Nocardiaceae*	CoA transferase A+B	
**WP_134475976.1**	*Cryobacterium tagatosivorans* Sr47	Actinomycetota, *Microbacteriaceae*		
WP_054814906.1	*Nocardia arizonensis* W9405 9405	Actinomycetota, *Nocardiaceae*	CoA transferase A+B	
WP_134571815.1	*Cryobacterium lyxosi* TMT1-1	Actinomycetota, *Microbacteriaceae*		
WP_020661493.1	*Amycolatopsis benzoatilytica* AK 16/65	Actinomycetota, *Pseudonocardiaceae*	CoA transferase A+B	
**WP_116765603.1**	*Arthrobacter humicola* JCM 15921	Actinomycetota, *Micrococcaceae*	CoA transferase A+B	
**WP_023985459.1**	*Mycolicibacterium neoaurum* DSM 1381	Actinomycetota, *Mycobacteriaceae*	CoA transferase A+B	
**WP_092108280.1**	*Cryobacterium luteum* CGMCC 1.11210	Actinomycetota, *Microbacteriaceae*		
WP_460993751.1	*Sinomonas soli* JCM 19961	Actinomycetota, *Micrococcaceae*	CoA transferase A+B	
WP_007269614.1	*Paeniglutamicibacter gangotriensis* Lz1y	Actinomycetota, *Micrococcaceae*	CoA transferase A+B	
WP_134503761.1	*Cryobacterium glaciale* HLT2-23	Actinomycetota, *Microbacteriaceae*		
WP_068398178.1	*Kribbia dieselivorans* NBRC 106261	Actinomycetota, *Intrasporangiaceae*		

WP_443020162.1	*Sinomonas cyclohexanicum* ATCC 51369	Actinomycetota, *Micrococcaceae*	(CoA transferase A+B)	
**WP_265998875.1**	*Mycobacterium pinniadriaticum* CVI	Actinomycetota, *Mycobacteriaceae*	CoA transferase A+B	
WP_218236902.1	*Nocardioides daeguensis* 2C1-5	Actinomycetota, *Nocardioidaceae*		
WP_035838931.1	*Cryobacterium roopkundense* DSM 21065	Actinomycetota, *Microbacteriaceae*		
WP_264069384.1	*Mycolicibacterium komossense* DSM 44078	Actinomycetota, *Mycobacteriaceae*		
WP_134173657.1	*Cryobacterium psychrophilum* DSM 4854	Actinomycetota, *Microbacteriaceae*		
WP_066585122.1	*Corynebacterium provencense* SN15	Actinomycetota, *Corynebacteriaceae*	CoA transferase A+B	
WP_129202879.1	*Xylanimonas allomyrinae* 2JSPR-7	Actinomycetota, *Promicromonosporaceae*	CoA transferase A+B	
WP_310051600.1	*Arthrobacter ginsengisoli* BE167	Actinomycetota, *Micrococcaceae*	CoA transferase A+B	
WP_036432990.1	*Mycolicibacterium margeritense* JCM 12375	Actinomycetota, *Mycobacteriaceae*		
WP_163751632.1	*Mycolicibacterium helvum* JCM 30396	Actinomycetota, *Mycobacteriaceae*		
WP_011280718.1	*Psychrobacter arcticus* 273-4	Gamma-Proteobacteria, *Moraxellaceae*		
WP_090363814.1	*Mycolicibacterium fluoranthenivorans* 2A	Actinomycetota, *Mycobacteriaceae*	CoA transferase A+B	
WP_085257429.1	*Mycobacterium saskatchewanense* JCM 13016	Actinomycetota, *Mycobacteriaceae*	CoA transferase	
**WP_167155922.1**	*Mycolicibacterium fluoranthenivorans* DSM 44556	Actinomycetota, *Mycobacteriaceae*	CoA transferase A+B	
**WP_023841948.1**	*Frankia casuarinae*	Actinomycetota, *Frankiaceae*	CoA transferase A+B	
**WP_233206156.1**	*Cryobacterium flavum* Hh8 C447	Actinomycetota, *Microbacteriaceae*		
WP_233551436.1	*Cryobacterium melibiosiphilum* CGMCC 1.11212	Actinomycetota, *Microbacteriaceae*		
WP_059038558.1	*Gordonia desulfuricans* 213E	Actinomycetota, *Gordoniaceae*	Phenylacetate-CoA ligase	
WP_241992823.1	*Cryobacterium frigoriphilum* Hh14	Actinomycetota, *Microbacteriaceae*		
**WP_011782042.1**	*Mycolicibacterium vanbaalenii* PYR-1	Actinomycetota, *Mycobacteriaceae*		
WP_062828849.1	*Mycolicibacterium brisbanense* JCM15654	Actinomycetota, *Mycobacteriaceae*		
**WP_067999595.1**	*Mycobacterium adipatum* YC-RL4	Actinomycetota, *Mycobacteriaceae*	CoA transferase A+B	
**WP_233195111.1**	*Cryobacterium levicorallinum* GMCC 1.11211	Actinomycetota, *Microbacteriaceae*		
WP_241976347.1	*Cryobacterium algoricola* MDB2-B	Actinomycetota, *Microbacteriaceae*		
WP_369045298.1	*Sinomonas puerhi* P10A9	Actinomycetota, *Micrococcaceae*	CoA transferase A+B	
**WP_213246495.1**	*Gordonia pseudamarae* BEN371	Actinomycetota, *Gordoniaceae*	CoA transferase A+B	
WP_066903612.1	*Millisia brevis* NBRC 105863	Actinomycetota, *Gordoniaceae*	CoA transferase A+B	
WP_228703023.1	*Marinobacter gelidimuriae* BF04_CF-4	Gamma-Proteobacteria, *Marinobacteraceae*		end of contig
WP_168581870.1	*Gephyromycinifex aptenodytis* NJES-13	Actinomycetota, *Dermatophilaceae*		
WP_198735606.1	*Corynebacterium marambiense* P5848 2	Actinomycetota, *Corynebacteriaceae*	CoA transferase A+B	
WP_006337001.1	*Gordonia rhizosphera* NBRC 16068	Actinomycetota, *Gordoniaceae*		
WP_026927734.1	*Granulicoccus phenolivorans* DSM 17626	Actinomycetota, *Propionibacteriaceae*		
**WP_151841987.1**	*Corynebacterium zhongnanshanii* zg-320	Actinomycetota, *Corynebacteriaceae*	CoA transferase A+B	
WP_035113253.1	*Corynebacterium auriscanis* DSM 44609	Actinomycetota, *Corynebacteriaceae*		
WP_069601453.1	*Brevibacterium aurantiacum* SMQ-1419	Actinomycetota, *Brevibacteriaceae*		

WP_006017763.1	*Helicobacter bizzozeronii* NHP20-010	Epsilon-Proteobacteria, *Helicobacteraceae*	CoA transferase A+B	
WP_041913084.1	*Helicobacter mustelae* NCTC12198	Epsilon-Proteobacteria, *Helicobacteraceae*		
WP_240377139.1	*Bacillus piscicola* FBU1786 8	Bacillota, *Bacillaceae*		
WP_051331125.1	*Aneurinibacillus terranovensis* DSM 18919	Bacillota, *Paenibacillaceae*	CoA transferase A+B	
WP_166910524.1	*Saccharococcus thermophilus* DSM 4749	Bacillota, *Anoxybacillaceae*		
WP_044894651.1	*Aeribacillus alveayuensis* 24KAM51	Bacillota, *Bacillaceae*		
WP_206338579.1	*Antarcticimicrobium sediminis* S4J41	Alpha-Proteobacteria, *Paracoccaceae*		
WP_048550690.1	*Nostocoides japonicum* T1-X7	Actinomycetota, *Intrasporangiaceae*		
WP_011941132.1	*Geotalea uraniireducens* Rf4	Desulfuromonadia, *Geobacteraceae*		
WP_265986281.1	*Ochrobactrum chromiisoli* YY2X	Alpha-Proteobacteria, *Brucellaceae*	CoA transferase A+B	
**WP_147179462.1**	*Paenirhizobium naphthalenivorans* NBRC 107585	Alpha-Proteobacteria, *Rhizobiaceae*	CoA transferase A+B	
**WP_227705594.1**	*Neorhizobium petrolearium* OS53 2	Alpha-Proteobacteria, *Rhizobiaceae*	CoA transferase A+B	
**WP_280924979.1**	*Ciceribacter sichuanensis* S101	Alpha-Proteobacteria, *Rhizobiaceae*	CoA transferase A+B	
WP_231711233.1	*Xanthobacter dioxanivorans* YN2	Alpha-Proteobacteria, *Xanthobacteraceae*		interrupted
WP_174523872.1	*Novosphingobium naphthalenivorans* NBRC 102051	Alpha-Proteobacteria, *Sphingomonadaceae*		
WP_175482105.1	*Gemmobacter aquatilis* DSM 3857	Alpha-Proteobacteria, *Paracoccaceae*		
WP_149576751.1	*Xanthobacter oligotrophicus* BF-7S	Alpha-Proteobacteria, *Xanthobacteraceae*		
WP_028094648.1	*Pseudodonghicola xiamenensis* DSM 18339	Alpha-Proteobacteria, *Paracoccaceae*		
WP_176832904.1	*Celeribacter baekdonensis* DSM 27375	Alpha-Proteobacteria, *Roseobacteraceae*		
WP_237347605.1	*Xanthobacter agilis* DSM 3770	Alpha-Proteobacteria, *Xanthobacteraceae*		
WP_100668997.1	*Kyrpidia spormannii* EA-1	Bacillota, *Alicyclonacillaceae*		
WP_221246912.1	*Acidocella aromatica* DSM 27026	Alpha-Proteobacteria, *Acidocellaceae*	CoA transferase A+B	
WP_144309479.1	*Denitromonas halophila* SFB-3 k141_79	Beta-Proteobacteria, *Zoogloeaceae*	(Acetoacetate-CoA ligase)	
WP_394035440.1	*Xanthobacter albus* V2C-8	Alpha-Proteobacteria, *Xanthobacteraceae*		
WP_236106558.1	*Zavarzinia marina* Adcm-6A	Alpha-Proteobacteria, *Zavarziniaceae*	Acetoacetate-CoA ligase	
WP_394093127.1	*Xanthobacter versatilis* V2C-9	Alpha-Proteobacteria, *Xanthobacteraceae*		
WP_244306982.1	*Azotobacter salinestris* KACC 13899	Gamma-Proteobacteria, *Pseudomonadaceae*	CoA transferase A+B	
WP_393997348.1	*Xanthobacter cornucopiae* V4C-4	Alpha-Proteobacteria, *Xanthobacteraceae*		
WP_393976680.1	*Xanthobacter nonsaccharivorans* 14g	Alpha-Proteobacteria, *Xanthobacteraceae*		
WP_228348559.1	*Flagellatimonas centrodinii* R2A-3	Gamma-Proteobacteria, *Nevskiacea*	Acetoacetate-CoA ligase	
WP_394104065.1	*Xanthobacter sediminis* V8C-5	Alpha-Proteobacteria, *Xanthobacteraceae*		
**WP_234052361.1**	*Xanthobacter variabilis* V4C-8	Alpha-Proteobacteria, *Xanthobacteraceae*		
WP_239017515.1	*Paenirhodobacter huangdaonensis* CGMCC 1.12963	Alpha-Proteobacteria, *Rhodobacter group*		
WP_206198906.1	*Zoogloea dura* G-4-1-14	Beta-Proteobacteria, *Zoogloeaceae*		
WP_173763567.1	*Nitrogeniibacter mangrovi* M9-3-2	Beta-Proteobacteria, *Zoogloeaceae*		
WP_237358978.1	*Allorhizobium borbori* DSM 26385	Alpha-Proteobacteria, *Rhizobiaceae*	CoA transferase A+B	
WP_109907261.1	*Zavarzinia aquatilis* HR-AS	Alpha-Proteobacteria, *Zavarziniaceae*	Acetoacetate-CoA ligase	

WP_211307312.1	*Sinimarinibacterium flocculans* CGMCC 1.10815	Gamma-Proteobacteria, *Nevskiacea*		interrupted
WP_147535170.1	*Bacillus marasmi* Marseille-P3556	Bacillota, *Bacillaceae*		
WP_245965265.1	*Paenirhodobacter hankyongi* BO-81	Alpha-Proteobacteria, *Rhodobacter group*		
WP_424193610.1	*Ampullimonas aquatilis* LMG 28208	Beta-Proteobacteria, *Alcaligenaceae*		
**WP_175555958.1**	*Azotobacter vinelandii* AEIV	Gamma-Proteobacteria, *Pseudomonadaceae*		
WP_090624836.1	*Azotobacter beijerinckii* DSM 373	Gamma-Proteobacteria, *Pseudomonadaceae*	CoA transferase A+B	
WP_240664546.1	*Paenirhodobacter ferrireducens* CCTCC AB 2012026	Alpha-Proteobacteria, *Rhodobacter group*		
WP_171294768.1	*Acinetobacter populi* PBJ7	Gamma-Proteobacteria, *Moraxellaceae*		
WP_093283829.1	*Solimonas aquatica* DSM 25927	Gamma-Proteobacteria, *Nevskiaceae*	Acetoacetate-CoA ligase	interrupted
WP_011800573.1	*Polaromonas naphthalenivorans* CJ2	Beta-Proteobacteria, *Comamonadaceae*		
WP_018990695.1	*Aromatoleum toluclasticum* ATCC 700605	Beta-Proteobacteria, *Rhodocyclaceae*		
WP_028008027.1	*Solimonas variicoloris* DSM 15731	Gamma-Proteobacteria, *Nevskiaceae*		
WP_420211148.1	*Burkholderia aenigmatica* ST2120	Beta-Proteobacteria, *Burkholderiaceae*	Acetoacetate-CoA ligase	
WP_170193164.1	*Gordonia asplenii* TBRC 11910 4	Actinomycetota, *Gordoniaceae*	Acetoacetate-CoA ligase	
WP_168147079.1	*Solimonas marina* C16B3	Gamma-Proteobacteria, *Nevskiaceae*	Acetoacetate-CoA ligase	interrupted
WP_020650917.1	*Solimonas variicoloris* DSM 15731	Gamma-Proteobacteria, *Nevskiaceae*	(Acetoacetate-CoA ligase)	interrupted
WP_200796690.1	*Lampropedia hyalina* DSM 16112	Beta-Proteobacteria, *Comamonadaceae*		
WP_198403510.1	*Celeribacter ethanolicus* TSPH2	Alpha-Proteobacteria, *Roseobacteraceae*		
WP_176870803.1	*Parasphingopyxis algicola* JCM 31719	Alpha-Proteobacteria, *Parapshingopyxidaceae*		
WP_007183975.1	*Hydrocarboniphaga effusa* AP103 245_1	Gamma-Proteobacteria, *Nevskiaceae*	Acetoacetate-CoA ligase	interrupted
WP_205424542.1	*Parageobacillus toebii* NEB718	Bacillota, *Anoxybacillaceae*		
WP_166253981.1	*Solimonas terrae* KIS83-12	Gamma-Proteobacteria, *Nevskiaceae*	Acetoacetate-CoA ligase	interrupted
WP_169259879.1	*Aromatoleum diolicum* 22Lin	Beta-Proteobacteria, *Rhodocyclaceae*		
WP_104230250.1	*Solimonas fluminis* HR-BB	Gamma-Proteobacteria, *Nevskiaceae*	Acetoacetate-CoA ligase	interrupted
WP_332290203.1	*Aquincola agrisoli* CGMCC 1.18515	Beta-Proteobacteria, *Sphaerotilaceae*	Acetoacetate-CoA ligase	
WP_091997889.1	*Paraburkholderia lycopersici* TNe-862	Beta-Proteobacteria, *Burkholderiaceae*	Acetoacetate-CoA ligase	interrupted
WP_208111900.1	*Zavarzinia compransoris* DSM 1231	Alpha-Proteobacteria, *Zavarziniaceae*	Acetoacetate-CoA ligase	
WP_013959294.1	*Cupriavidus necator* ATCC 43291	Beta-Proteobacteria, *Burkholderiaceae*	Acetoacetate-CoA ligase	interrupted
**WP_071010558.1**	*Cupriavidus malaysiensis* USMAA1020	Beta-Proteobacteria, *Burkholderiaceae*	Acetoacetate-CoA ligase	
**WP_011354810.1**	*Burkholderia lata* DSM 23089	Beta-Proteobacteria, *Burkholderiaceae*	Acetoacetate-CoA ligase	
WP_029921399.1	*Nevskia soli* DSM 19509	Gamma-Proteobacteria, *Nevskiaceae*	Acetoacetate-CoA ligase	interrupted
WP_078427661.1	*Alkalihalobacterium alkalinitrilicum* DSM 22532	Bacillota, *Bacillaceae*		
WP_142494258.1	*Thalassovita litoralis* DSM 29506	Alpha-Proteobacteria, *Roseobacteraceae*		
WP_184064027.1	*Sphingosinicella soli* DSM 17328	Alpha-Proteobacteria, *Sphingosinicellaceae*		
WP_012110036.1	*Parvibaculum lavamentivorans* DS-1	Alpha-Proteobacteria, *Parvibaculaceae*	Acetoacetate-CoA ligase	
WP_180284774.1	*Azospirillum oleiclasticum* ROY-1-1-2	Alpha-Proteobacteria, *Azospirillaceae*		

WP_036862101.1	*Porticoccus hydrocarbonoclasticus* MCTG13d	Gamma-Proteobacteria, *Porticoccaceae*		
WP_305169416.1	*Porticoccus litoralis* DSM 25064	Gamma-Proteobacteria, *Porticoccaceae*		
**WP_054818571.1**	*Paenibacillus naphthalenovorans* 32O-Y	Bacillota, *Paenibacillaceae*	Phenylacetate-CoA ligase	
WP_194213009.1	*Kordiimonas pumila* N18	Alpha-Proteobacteria, *Kordiimonadaceae*		
WP_405054185.1	*Thalassotalea psychrophila* SQ149	Gamma-Proteobacteria, *Cowelliaceae*	Acetoacetate-CoA ligase	
WP_011809591.1	*Verminephrobacter eiseniae* EF01-2	Beta-Proteobacteria, *Comamonadaceae*		
WP_022960593.1	*Spongiibacter tropicus* DSM 19543	Gamma-Proteobacteria, *Spongiibacteraceae*	Acetoacetate-CoA ligase	
WP_198569439.1	*Spongiibacter nanhainus* csc3.9	Gamma-Proteobacteria, *Spongiibacteraceae*	Acetoacetate-CoA ligase	
WP_320669488.1	*Patulibacter defluvii* DM4	Actinomycetota, *Patulibacteraceae*		
**WP_254824572.1**	*Haloglomus salinum* ZY58	Archaea, *Halobacteria*		
WP_219042113.1	*Zhongshania aquimaris* CAU 1632	Gamma-Proteobacteria, *Spongiibacteraceae*	Acetoacetate-CoA ligase	
WP_007569898.1	*Patulibacter medicamentivorans* I11	Actinomycetota, *Patulibacteraceae*		
WP_010283668.1	*Bacillus timonensis* MGYG-HGUT-01405	Bacillota, *Bacillaceae*	Phenylacetate-CoA ligase	
WP_246989256.1	*Halorientalis marina* GDY88 [plasmid]	Archaea, *Halobacteria*		
WP_021690176.1	*Caenibius tardaugens* NBRC 16725	Alpha-Proteobacteria, *Erythrobacteraceae*		
WP_269620706.1	*Zhongshania aquatica* BJYM1	Gamma-Proteobacteria, *Spongiibacteraceae*	Acetoacetate-CoA ligase	
WP_338448811.1	*Niallia oryzisoli* DSM 29761	Bacillota, *Bacillaceae*		
WP_099364373.1	*Fredinandcohnia onubensis* 0911MAR22V3	Bacillota, *Bacillaceae*	Phenylacetate-CoA ligase	
WP_013677933.1	*Pseudonocardia dioxanivorans* CB1190	Actinomycetota, *Pseudonocardiaceae*	Phenylacetate-CoA ligase	
WP_259314056.1	*Capillimicrobium parvum* 0166_1	Actinomycetota, *Capillimicrobiaceae*		
WP_112425605.1	*Thermogemmatispora tikiterensis* T81	Ktedonobacteria, *Thermogemmatisporaceae*	Phenylacetate-CoA ligase	
WP_133881603.1	*Panacagrimonas perspica* DSM 26377	Gamma-Proteobacteria, *Nevskiaceae*		
WP_346091545.1	*Pseudonocardia benzenivorans* JCM 12694	Actinomycetota, *Pseudonocardiaceae*	Phenylacetate-CoA ligase	
WP_412898957.1	*Bacillus jepli* 1P02SD	Bacillota, *Bacillaceae*	Phenylacetate-CoA ligase	
WP_198884416.1	*Acuticoccus mangrovi* B2012	Alpha-Proteobacteria, *Amorphaceae*		
**WP_196426827.1**	*Paenibacillus validus* S1	Bacillota, *Paenibacillaceae*	Phenylacetate-CoA ligase	
WP_343982774.1	*Pseudonocardia aurantiaca* JCM 11838	Actinomycetota, *Pseudonocardiaceae*	Phenylacetate-CoA ligase	
WP_028066683.1	*Solirubrobacter soli* DSM 22325	Actinomycetota, *Solirubrobacteraceae*	Acetoacetate-CoA ligase	
WP_147101802.1	*Pseudonocardia sulfidoxydans* JCM 10411 tig00000001	Actinomycetota, *Pseudonocardiaceae*	Phenylacetate-CoA ligase	
WP_232872601.1	*Pseudonocardia kujensis* KCTC 29062	Actinomycetota, *Pseudonocardiaceae*	Phenylacetate-CoA ligase	
WP_377713717.1	*Paraburkholderia denitrificans* KACC 12733	Beta-Proteobacteria, *Burkholderiaceae*	Acetoacetate-CoA ligase	
WP_132877455.1	*Tamaricihabitans halophyticus* DSM 45765	Actinomycetota, *Pseudonocardiaceae*		
WP_206808828.1	*Paradesulfitobacterium ferrireducens* PLL0 L1_ctg0002	Bacillota, *Peptococcaceae*	Phenylacetate-CoA ligase	
WP_337702696.1	*Actinomycetospora flava* OC33-EN07 3	Actinomycetota, *Pseudonocardiaceae*		
WP_139940270.1	*Emcibacter nanhaiensis* MCCC 1A06723 6	Alpha-Proteobacteria, *Emcibacteraceae*		
WP_091675330.1	*Amycolatopsis marina* CGMCC 4.3568	Actinomycetota, *Pseudonocardiaceae*		
WP_218600732.1	*Pseudonocardia abyssalis* KRD168	Actinomycetota, *Pseudonocardiaceae*	Phenylacetate-CoA ligase	

WP_459997565.1	*Paradesulfitobacterium aromaticivorans* JCM 15765	Bacillota, *Peptococcaceae*	Phenylacetate-CoA ligase	
WP_132423221.1	*Pseudonocardia endophytica* DSM 44969	Actinomycetota, *Pseudonocardiaceae*	Phenylacetate-CoA ligase	
WP_091803717.1	*Prauserella marina* DSM 45268	Actinomycetota, *Pseudonocardiaceae*		
WP_316510085.1	*Mycobacterium wendilense* Mu0050	Actinomycetota, *Mycobacteriaceae*		
WP_454559992.1	*Parapedomonas caeni* M-36	Alpha-Proteobacteria, *Sphingosinicellaceae*		
WP_191249990.1	*Kordiimonas sediminis* KCTC 42590	Alpha-Proteobacteria, *Kordiimonadaceae*		
WP_077116735.1	*Nocardia donostiensis* W9944 3	Actinomycetota, *Nocardiaceae*		
WP_022977952.1	*Nevskia ramosa* DSM 11499	Gamma-Proteobacteria, *Nevskiaceae*		interrupted
WP_226952659.1	*Mesopusillimonas faecipullorum* CC-YST705	Beta-Proteobacteria, *Alcaligenaceae*		
WP_311657189.1	*Spectribacter acetivorans* P385 2	Gamma-Proteobacteria, *Salinisphaeraceae*	Phenylacetate-CoA ligase	
WP_028321587.1	*Desulfatiglans anilini* DSM 4660	Desulfobacteria, *Desulfatiglandaceae*	Phenylacetate-CoA ligase	
**WP_197719750.1**	*Pseudonocardia autotrophica* DSM 535	Actinomycetota, *Pseudonocardiaceae*		
WP_011237169.1	***Aromatoleum aromaticum* EbN1**	Beta-Proteobacteria*, Rhodocyclaceae*	Benzoylacetate-CoA ligase	
WP_460751741.1	*Nocardiopsis oceani* JCM 31824	Actinomycetota, *Nocardiopsidaceae*		
WP_096449779.1	*Thauera sinica* K11	Beta-Proteobacteria, *Zoogloeaceae*		
WP_436348269.1	*Natronorubrum halobium* FCH18a [plasmid]	Archaea, Halobacteria, *Natrialbaceae*	Phenylacetate-CoA ligase	
WP_169203719.1	*Aromatoleum bremense* PbN1	Beta-Proteobacteria, *Rhodocyclaceae*	Benzoylacetate-CoA ligase	
WP_345414753.1	*Actinomycetospora chlora* JCM 17979	Actinomycetota, *Pseudonocardiaceae*		
**WP_344017861.1**	*Pseudonocardia kongjuensis* JCM 11896	Actinomycetota, *Pseudonocardiaceae*		
WP_222047687.1	*Modestobacter lapidis* JCM 31703	Actinomycetota, *Geodermatophilaceae*		
WP_344687998.1	*Blastococcus jejuensis* JCM 15614	Actinomycetota, *Geodermatophilaceae*		
WP_220635798.1	*Georgfuchsia toluolica* G5G6	Beta-Proteobacteria, *Sterolibacteriaceae*		
WP_183338116.1	*Conexibacter arvalis* DSM 23288	Actinomycetota, *Conexibacteraceae*		
WP_028320070.1	*Desulfatiglans anilini* DSM 4660	Desulfobacteria, *Desulfatiglandaceae*	Phenylacetate-CoA ligase	
WP_459883930.1	*Halostagnicola bangensis* JCM 18750	Archaea, Halobacteria, *Natrialbaceae*	Phenylacetate-CoA ligase	
WP_311653569.1	*Spectribacter hydrogenoxidans* W335 12	Gamma-Proteobacteria, *Salinisphaeraceae*	Phenylacetate-CoA ligase	
WP_180900617.1	*Martelella soudanensis* NC20	Alpha-Proteobacteria, *Aurantimonadaceae*		
WP_308478266.1	*Mycobacterium burgundiense* Mu0053	Actinomycetota, *Mycobacteriaceae*		
WP_311172080.1	*Halobellus ordinarius* ZY16	Archaea, Halobacteria, *Haloferacaceae*	Phenylacetate-CoA ligase	
WP_226777012.1	*Haloterrigena alkaliphila* KZCA68	Archaea, Halobacteria, *Natrialbaceae*	Phenylacetate-CoA ligase	
WP_381485382.1	*Sphingosinicella xenopeptidilytica* CCUG 52537	Alpha-Proteobacteria, *Sphingosinicellaceae*		
WP_121534466.1	*Paenirhodobacter hankyongi* BO-81	Alpha-Proteobacteria*, Rhodobacter group*		
WP_313684291.1	*Halobellus marinus* DFY28	Archaea, Halobacteria, *Haloferacaceae*	Phenylacetate-CoA ligase	
WP_268758350.1	*Amycolatopsis cynarae* HUAS 11-8	Actinomycetota, *Pseudonocardiaceae*	(Phenylacetate-CoA ligase)	
WP_128156706.1	*Paenirhodobacter hankyongi*	Alpha-Proteobacteria, *Rhodobacter group*		
WP_284307557.1	*Hydrogenophaga electricum* NBRC 109341	Beta-Proteobacteria, *Comamonadaceae*	Phenylacetate-CoA ligase	
WP_169595442.1	*Rhizobium terricola* S-51	Alpha-Proteobacteria, *Rhizobiaceae*		
WP_248960971.1	*Sphaerisporangium perillae* NEAU-ZS1	Actinomycetota, *Streptosporangiaceae*		

WP_144586298.1	*Amycolatopsis rhizosphaerae* TBRC 6029	Actinomycetota, *Pseudonocardiaceae*		
WP_133359705.1	*Antarcticimicrobium luteum* 318-1	Alpha-Proteobacteria, *Paracoccaceae*		
**WP_092816955.1**	*Halopenitus malekzadehii* IBRC-M10418	Archaea, Halobacteria*, Haloferacaceae*	Phenylacetate-CoA ligase	interrupted
WP_096389760.1	*Halopenitus persicus* CBA1233	Archaea, Halobacteria, *Haloferacaceae*	Phenylacetate-CoA ligase	
WP_121053301.1	*Sphingosinicella microcystinivorans* B-9	Alpha-Proteobacteria*, Sphingosinicellaceae*		
WP_132830415.1	*Antarcticimicrobium sediminis* S4J41	Alpha-Proteobacteria, *Paracoccaceae*		
**WP_258937562.1**	*Prauserella flava* DSM 45265	Actinomycetota, *Pseudonocardiaceae*	Phenylacetate-CoA ligase	
WP_109773704.1	*Quadrisphaera granulorum* DSM 44889	Actinomycetota, *Kineosporiaceae*	Phenylacetate-CoA ligase	
WP_345421382.1	*Pseudonocardia xishanensis* JCM 17906	Actinomycetota, *Pseudonocardiaceae*		

Supplementary Table 8: Results of sequencing across the modified gene locus of the Δ*acrE* deletion mutant. Mismatches are highlighted in red.

Sequencing result #1

ACGGGTTCCGCCAGGGTCTCGGCCAAGGTCGTCGTGAGCGGGGCGGGCATGGCCTGGATGTTGCGCAGCACGTACAG
GGTGGGGTTGGTGGAGGACACCGTGAGCCCGGACAGGTCGGTCTCGCTCCCGTCCACGAGGATGCTCCGCCCCCCGG
CGTACAGGGAGTGGAAGATCTCCGACGCGAGAGACGCCTTCCCCGTGCCCGGTTCGCCGGTGAGGAGCAACTTCGCC
CCGGCGGACAGGGCCTCCTCGATCTCGTTGCAGGCGCGCATCCACAGAGCGGAGGAACCGCTCGAGCGCAGACGAT
CGGTGCCGGCAGCGGCCAGACCGACCCGCCCGGAGTGCAGCCGGTGCTCGCGGACCCGGTTCGGAACGGACGGAAG
CGGTGCCGCGGCCGCGGCCGCCTCGGAGACCTCGACGAGGTATCCGGCGGTGTCCTCGCCCGCCAGGATCCGGGTCG
CGCGAACCCGGACGGACAGGCCGGTGCCCAGCTCGACCAGTTCGTCGATGCGCGCGCGGTGCCCGATGACGTAGTCG
ACGTGTTCACCGACACGGCGTTGGTCCTCCGGCGCGAGGAGGGTGTGGGCGAGCGGATTCGCGAGCGACATCGCGCT
CCCGATCGCCATGAGGGCCGTGCCGCCGCGAGCGTCGGCCCGCACGAACGCGTCGAACAGCGCGTGCTGGCGGGCG
CTCCGGTCCCGGAACAGATTCTGTTCGATCTCCGCCGCCGCGGTCCGCACGACGGTCGTGAGCAGCGGCGAGGAATG
CTCGGTGAGGCAACTGATGTCGATGACGCCGGCGACGCGTCCGCTGAACGGGTCCCGGATCGGCGCCCCGGAGCAG
GCGAACGGCTGGAACCGTTCGTGGAAGTGCTCGGCGCCCTCGATCTGCACCGGGCGCCCGGATTCCAGCACGGTGCC
GACGCCGTTGGTGCCGATCTGCCCCTCGCAGTAGTCGAATCCCGGCGCGAGGGAGACATGGTCGAGCAACCGTCCGA
TGGCGCGGGCCCCGTCGAGGCGCATCAGCACCTGTGCCTTGGCGTCGGTCAGGGCGATGCTCACCGGGACGTCGACG
GTCTCCTCCGCGAGGCGGTCCAGGACCGGCAGCGAGCACTGGACCAGCCGAGAGGTCCGGTCCMCCTCCCCCGCGA
AC

Sequencing result #2

CCAACATCAGCCGGACTCCGATTACCTCGGGAACTTGCTCCGTAGTAAGACATTCATCGCGCTTGCTGCCTTCGACCA
AGAAGCGGTTGTTGGCGCTCTCGCGGCTTACGTTCTGCCCAAGTTTGAGCAGCCGCGTAGTGAGATCTATATCTATGA
TCTCGCAGTCTCCGGAGAGCACCGGAGGCAGGGCATTGCCACCGCGCTCATCAATCTCCTCAAGCATGAGGCCAACG
CGCTTGGTGCTTATGTGATCTACGTGCAAGCAGATTACGGTGACGATCCCGCAGTGGCTCTCTATACAAAGTTGGGCA
TACGGGAAGAAGTGATGCACTTTGATATCGACCCAAGTACCGCCACCTAACAATTCGTTCAAGCCGAGATCGGCTTC
CCGGCCGCGGAGTTGTTCGGTAAATTGTCACAACGCCGCCACAAGAGTGCCGGGGCGAGGCGCCCGATGAAGAGCG
CGGACCGACGGCTGCGGGTGGCGGTCGCCCGGGCCGGATTCCTCGACTCCGGCGAACCGGACGATTCGCTGGTCTCC
TCTGTCGTGTCCGCGTCGTGGCGGCGCTCCGCCGCGGCGGGCGTGCGACCGTCCGGTGGGGAGGTCCGGTTCGCGGG
GGAGGTGGACCGGACCTCTCGGCTGGTCCAGTGCTCGCTGCCGGTCCTGGACCGCCTCGCGGAGGAGACCGTCGACG
TCCCGGTGAGCATCGCCCTGACCGACGCCAAGGCACAGGTGCTGATGCGCCTCGACGGGGCCCGCGCCATCGGACGG
TTGCTCGACCATGTCTCCCTCGCGCCGGGATTCGACTACTGCGAGGGGCAGATCGGCACCAACGGCGTCGGCACCGT
GCTGGAATCCGGGCGCCCGGTGCAGATCGAGGGCGCCGAGCACTTCCACGAACGGTTCCAGCCGTTCGCCTGCTCCG
GGGCGCCGATCCGGGACCCGTTCAGCGGACGCGTCGCCGGCGTCATCGACATCAGTTGCCTCACCGAGCATTCCTCG
CCGCTGCTCACGACCGTCGTGCGGACCGCGGCGGCGGAGATCGAACAGAATCTGTTCCGGGACCGGAGCGCCCGCCA
GCACGCGCTGTTCGACGCGTTCGTGCGGGCCGACGCTCGCGGCGGCACGGCCCTCATGGCGATCGGGAGCGCGATGT
CGCTCGSGAAT-CGCTCGCC--MMCCCTCCTC-SSCCGGAGGACC-ACGCCGTGTCGG-GAAC-CGT

Sequencing result #3

CYAKGGCTTCGGCAAGTACCGCGGCGGCATGGGCTACGAGATGATCGTGGCGGCCGAGGGCACCCCGCTGTGGGGC
TTCATGACGGTGACCTCCGGTGCCAAGTTCTCCTCCATCAGCGGACAGTTCGGCGGGTACGGCTGCAACACCTACCCG
CTCGCGACGGTCAAGGGCGTCAACGTCTACGACGAGCTGCGCCGCAACCCGGAACGGTTCGACCTGTCCATCGAGAA
GGTGATGAACGAACAGCCCTTCGAGGGCGCCGAGTACAGCACCAGCCACATGGGTCTGCAGTTCGACGTCGCCAAG
GACGGTGAGCTGTACATGATTTCGCAGGGCACGGGCGGAGGGTACGGAGATCCGCTCGAACGCGCCCCGGAGGACG
TGGTCGCGGATCTCGAGATCGACCGGATCTCCGCGAAGGTGGCGCACGACATCTACGGCGTCGTGTACGCGCCGGAC
ACGTTCGTCGTGGACGTCGACGCCACGGAGACGCTACGCGCGTCGATGCGGCGCGACCGGCTCTCCCGCGGTGTGCC
CTACGGCGAGTTCGTCGAGAAGTTCGTGACGCCCGAACCTCCGCAGGACCTGCTGTACTACGGATCCTGGGGCGACA
CCGAGGAACTGACCGCCACGCACTTCACCGTGCACGGCGCCGAGCGGGTCACCACGACGATCGACAAGATGCCGAT
CATCATGATGCCCGACCGGCGGGAGCTGAAGATCGCCGAGCTCACGGCCCGGGTGAAGGAACTCGAGTCCAAGCAC
GGCGAGATCGTCCACCGCAAGTCCTGAAACCGGTCCGGCCGGAGCCGGTTCCGGCCGGCTCCGGCCACCGGAT-CAC
CAARGCCGGGTGGCCCGCACGTGTGCTCCCCTCACCGTGCGGGCACCCACTCCCTGGAGTCAGAAACATGACCATCT
CGTTTCCCGCCGTACTCGACGCGCCGGTCTCCGGGCGCCGGGTCCCGCTCGTCGTCGACCATCTCGACTACAGCCGCC
GGGTCCTGCTCCGCGGCAACCCGGTGCCCTGGGAGGATCCCACCGCGCTGTCGAACTTCCTCAACCAGG-CCACGGGC
TGCTGCGACCGGACGTCACGCTGCTCGATCTCGGCGAGTACTGACGCMCACCGTGGAAACGGATGAAGG-MCGAACC
CAGTTGAC-TAAGCCTGTTCGGTTCGT-AACTGTAATGCAAGTAGCGT

Supplementary Table 9: Search parameters for interpretation of mass spectrometric data with Proteome Discoverer 1.4.

Enzyme Name	Trypsin (Full)
Max. Missed Cleavage Sites	2
Min. Peptide Length	7
Max. Peptide Length	144
Precursor Mass Tolerance	10 ppm
Fragment Mass Tolerance	0.02 Da
Max. Equal Modifications Per Peptide	8
Max. Dynamic Modifications Per Peptide	4
1. Dynamic Modification	Oxidation / +15.995 Da (M)
2. Dynamic Modification	Deamidation /+0.984 Da (N, Q)

Supplementary Table 10: Optimized LC-MS parameter settings for acetoacetate determination.

Analyte	Acetoacetate (Quantifier)	Acetoacetate (Qualifier)
**Precursor Ion [m/z]**	101	101
**Product Ion [m/z]**	57.1	101
**Dwell Time [ms]**	40	40
**Fragmentor Voltage [V]**	380	380
**Collision Energy [eV]**	8	0
**Cell Accelerator Voltage [V]**	5	5
**Polarity**	Negative	Negative
